# Quantitative modeling of the physiology of ascites in portal hypertension

**DOI:** 10.1186/1471-230X-12-26

**Published:** 2012-03-27

**Authors:** David G Levitt, Michael D Levitt

**Affiliations:** 1 Department of Integrative Biology and Physiology, University of Minnesota, 6-125 Jackson Hall, 321 Church St. S. E., Minneapolis, MN 55455, USA; 2 Research Service, Veterans Affairs Medical Center, VAMC/111D, 1 Veterans Drive, Minneapolis, MN 55417, USA

**Keywords:** Ascites, Cirrhosis, Portal hypertension, Wedge pressure

## Abstract

Although the factors involved in cirrhotic ascites have been studied for a century, a number of observations are not understood, including the action of diuretics in the treatment of ascites and the ability of the plasma-ascitic albumin gradient to diagnose portal hypertension. This communication presents an explanation of ascites based solely on pathophysiological alterations within the peritoneal cavity. A quantitative model is described based on experimental vascular and intraperitoneal pressures, lymph flow, and peritoneal space compliance. The model's predictions accurately mimic clinical observations in ascites, including the magnitude and time course of changes observed following paracentesis or diuretic therapy.

## Background

Ascites commonly is regarded as a clinical condition that can be understood in terms of classic physiological principals. The fundamental factors involved in the formation of ascites were established over a century ago when Starling [1] used observations of thoracic duct lymph flow and fluid absorption from the peritoneal space to support his classic description of the forces that determine capillary fluid balance.

Over the past 30 years, ascites research largely has focused on associated systemic abnormalities such as increases in cardiac output, blood volume, renal sodium retention and reduction in total systemic vascular resistance. Whether these systemic abnormalities are the effect or cause of ascites has been controversial. Rocco and Ware [2] review the two older competing hypotheses: 1) the "underfill" theory in which ascites formation is the primary event causing the systemic changes, versus 2) the "overflow" theory in which renal sodium retention is the primary event. Recent reports [3-9], which have emphasized the renal and systemic effects of vasoactive compounds such as nitric oxide and the "hyperdynamic syndrome", favor the "forward" theory, which represents a synthesis of the underfill and overflow theories.

This focus on renal and systemic effects has led investigators to lose sight of the local factors within the peritoneal cavity that actually are responsible for the accumulations of ascites. For example, a recent review [10] of ascites summarized the pathogenesis as follows:

"The most acceptable theory for ascites formation is peripheral arterial vasodilation leading to underfilling of circulatory volume. This triggers the baroreceptor-mediated activation of renin-angiotensin-aldosterone system, sympathetic nervous system and nonosmotic release of vasopressin to restore circulatory integrity. The result is an avid sodium and water retention, identified as a preascitic state. This condition will evolve in overt fluid retention and ascites, as the liver disease progresses. Once ascites is present, most therapeutic modalities are directed on maintaining negative sodium balance, including salt restriction, bed rest and diuretics."

How the above described systemic changes translate into ascites accumulation is poorly understood at the quantitative level, and, in fact, is seldom discussed (as illustrated by the above quote). As a result, several clinically important observations remain unexplained. For example, how do the systemic alterations induced by salt restriction and diuretics lead to a reduction in ascites [11] and why does the commonly employed measurement of plasma-ascitic fluid albumin gradient accurately diagnose portal hypertension. In an attempt to better understand the physiology of ascites, we present what appears to be the first quantitative model of the formation and removal of ascitic fluid. In this model, ascites accumulation is explained solely by pathophysiological alterations within the peritoneal cavity; systemic abnormalities are considered relevant only to the extent that they alter intraperitoneal physiology. Insights derived from this model may provide answers to previously unexplained clinical observations in patients with ascites.

Section I of the Discussion provides a brief quantitative description of the factors involved in fluid balance in the peritoneal space of normal subjects. Section II presents an analysis of the pathological abnormalities in the peritoneal cavity that are involved in the accumulation of ascites. This discussion includes mechanistic explanations for several clinically important, but poorly understood phenomena. Finally, in Section III, using a reasonable set of assumptions, a quantitative physical model describing ascites accumulation is developed and discussed. This model accurately predicts the magnitude and time course of the changes in ascites volume that are observed clinically with diuretic treatment or paracentesis. In sections I-III we have focused on the key factors and main supporting experimental results. The attached Additional file 1 (Section A), provides a more detailed discussion of the evidence supporting (or contradicting) the model's assumptions.

## Discussion

### I. Physiological model of normal peritoneal fluid balance

**Figure 1:**
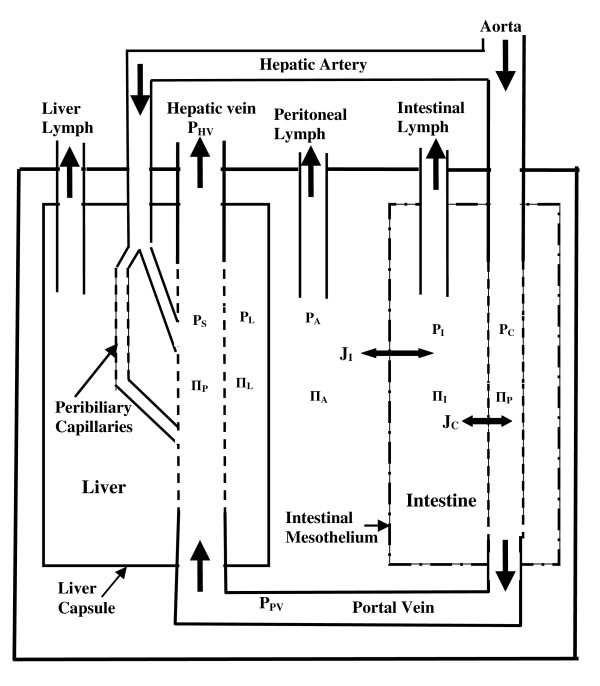
**Schematic diagram of the peritoneal space**. The "intestine" represents all non-hepatic, intraperitoneal organs (including the mesentery). The symbols P and Π indicate the hydrostatic and colloid osmotic pressure of the different compartments. See "Notation" for a definition of all the terms.

A schematic diagram of the peritoneal space is shown in Figure 1. Two compartments exchange with this space, the liver and the "intestine". The "intestine" is assumed to represent all non-hepatic, intraperitoneal organs (including the mesentery). The liver has a dual blood flow--the portal vein and the hepatic artery. One branch of the hepatic artery (about 2/3 of flow) directly connects to the sinusoids and the other branch supplies the peribiliary capillaries of the bile ducts which then drain into the sinusoids [12]. The intestinal capillary and liver sinusoidal colloid osmotic pressures are assumed to equal the systemic plasma colloid osmotic pressure (Π_P_).

#### A. Fluid transport across the intestinal capillaries

Fluid balance across the intestinal capillaries is a function of the hydrostatic and colloid osmotic pressures. Since the intestinal capillary membrane is relatively impermeable to protein, the volume flux between the capillary and tissue space (J_C_) is described by Starling's relation:

(1) $$J_{C}=L_{C}\left[\left(P_{C}-P_{I}\right)-\left(\Pi_{P}-\Pi_{I}\right)\right]$$

where J_C _is the flux across the capillary, L_C _is the capillary hydraulic permeability, and P_C _and P_I _are the hydraulic pressures in the capillary and tissue and Π_P _and Π_I _are the oncotic pressures in the capillary and tissue. The capillary membrane has a slow diffusional leak of protein (primarily albumin). In the normal steady state, this slow leak is balanced by protein removal from the tissue space by the "intestinal" lymph flow, and the interstitial protein concentration is roughly 70% of the plasma concentration [13]. Because the hydraulic permeability is large compared to this slow leak, to a first approximation J_C _≈ 0 and the difference in hydraulic pressure across the capillary equals the oncotic pressure difference:

(2) $$\Pi_{P}-\Pi_{I}=P_{C}-P_{I}$$

As discussed in the next section, P_I _is approximately equal to P_A _(intra-abdominal pressure) so that:

(3) $$\Pi_{I}\;\approx \;\;\Pi_{P}\;+P_{A}\;-P_{C}$$

Normally, the plasma colloid osmotic pressure (Π_P_) is about 25 mm Hg and the supine intra-abdominal pressure (P_A_) is about 2 mm Hg [14]. Although direct experimental measurement of the intestinal hydrostatic capillary (P_C_) and tissue colloid osmotic (Π_I_) pressures are difficult and uncertain (see Additional file 1 Section IA for more details), an estimate of the normal values [15] are P_C _= 9 and Π_I _= 18 mm Hg, which are consistent with eq. (3). This is a dynamic equilibrium. If, for example, capillary pressure (P_C_) is raised (e.g., by constricting the portal vein), there will be an initial increase in capillary filtration (J_C_, eq. (1)) increasing intestinal lymph flow and "washing down" the tissue protein (Π_I_) until a new steady state relation (eq. (3)) is established.

#### B. Fluid transport across the intestinal mesothelium

The mesothelial membrane (Figure 1) separates the intestinal tissue space from the peritoneal space. It is assumed that the "intestinal" mesothelial and capillary membrane have similar protein permeability properties, with the rate of fluid exchange between the tissue and peritoneal space (J_I_) described by:

(4) $$J_{I\;}\;=\;L_{I}\left[\left(P_{I}-P_{A}\right)\;-\;\left(\Pi_{I}-\Pi_{A}\right)\right]$$

where P_A _and Π_A _are the hydrostatic and colloid osmotic pressures in the peritoneal (ascitic) space and L_I _is the mesothelial hydraulic permeability. It is also assumed that the mesothelial membrane cannot support appreciable mechanical forces so that, to a first approximation, there is no hydrostatic pressure gradient across this membrane:

(5) $$P_{I}\;=\;\;P_{A}$$

and eq. (4) reduces to:

(6) $$J_{I\;}\;=\;L_{I}\left[\;\Pi_{A}-\Pi_{I}\right]$$

As discussed in detail in the Additional file 1 Section IB, the mesothelial barrier is ignored in the peritoneal dialysis literatures where it is assumed that this membrane is highly permeable to both protein and fluid. This assumption would not significantly change any of our model results or conclusions and, as is shown Section III, the quantitative steady state relations are identical, with or without, a mesothelial barrier. We feel experimental evidence supporting a significant mesothelial barrier is sufficient to keep this barrier in the model.

#### C. Fluid transport across the liver

The fenestrated liver sinusoids allow relatively free passage of proteins between the blood and tissue [16]. Thus, to a first approximation, the sinusoidal and tissue hydrostatic [17] and colloid osmotic pressures [18] are approximately equal:

(7) $$P_{L}\;\approx \;P_{S}\;\;\;\;\;\;\;\;\;\;\;\;\;\;\;\;\;\;\;\;\;\;\;\;\;\;\;\;\Pi_{L}\;\approx \;\;\Pi_{P}$$

The normal liver sinusoidal pressure (P_S_) and, therefore, the liver tissue pressure (P_L_) is about 5 mm Hg (see Section IIC), significantly greater than the normal pressure in the peritoneal space (P_A _≈ 2 mmHg). It follows that the liver capsule, in contrast to the intestinal mesothelium, is able to maintain a hydrostatic pressure difference between the tissue and peritoneal space. Assuming that the capsule is relatively impermeable to albumin, the pressure difference between the liver tissue and peritoneal space (P_L_-P_A_) would have to overcome the tissue colloid osmotic pressure (≈ 25 mmHg) before there would be net fluid movement across the liver surface. As is discussed below, fluid leaks from the liver into the peritoneal space at pressures well below 25 mm Hg indicating that the source of this fluid is rupture of either the capsule or surface lymphatics.

#### D. Fluid exchange in the peritoneal space

Normal fluid movement between the peritoneal space and the systemic circulation is a combination of the "intestinal" mesothelium (J_I_) flux and the peritoneal lymph drainage (as discussed above, liver fluid flux is normally low). Since the mesothelial hydraulic permeability (L_I_) should be relatively large compared to the normal small lymph flow, to a first approximation J_I _= 0 and, from eq. (6), the peritoneal (Π_A_) and the "intestinal" (Π_I_) colloid osmotic pressures are equal:

(8) $$\Pi_{A}\;\approx \;\Pi_{I}$$

The peritoneal lymph drainage (Figure 1) removes fluid in bulk, with no sieving of the peritoneal protein concentration [19,20]. Our limited understanding of the rate of peritoneal lymph flow and its dependence on intra-abdominal pressure and volume is discussed in more detail in Section IID.

### II. Factors involved in the formation of Ascites in portal hypertension

#### A. Increase in portal vein pressure

In the absence of increased hepatic sinusoidal pressure, marked elevations in portal vein pressure are required to induce ascites. Moderate increases in portal pressure induced experimentally via portal vein constriction [21] or clinically by portal vein thrombosis [22] do not result in appreciable ascites. This observation is explained by the above model. The peritoneal cavity normally contains a small amount of fluid (up to 100 ml) that is in osmotic equilibrium with intestinal tissue (eq. (8)). A rise in portal pressure elevates intestinal capillary pressure, increasing capillary filtration (J_C_, eq. (1)) and intestinal lymph flow. This washes out tissue protein, and the tissue colloid osmotic pressure (Π_I_) falls until a new steady state (eq. (2)) is established. This decrease in Π_I _produces an osmotic water flux from intestinal tissue into the peritoneal space (eq. (6)), diluting the peritoneal protein until a new steady state is reached in which peritoneal colloid osmotic pressure equals the low value in the intestinal tissue (eq. (8)). This initial increase in peritoneal (ascitic) volume is small. For example, a reduction of tissue protein by a factor of two would result in a doubling of the initial small peritoneal volume. The lymphatic drainage of the peritoneal cavity brings the peritoneal volume back towards its original volume. The important point is that a new equilibrium will be rapidly established in which the volume flux between the intestine and peritoneal space is zero at the increased portal and intestinal capillary pressure, and the elevation in portal pressure does not produce appreciable ascites. This dynamic equilibrium reaches its limit when the intestinal tissue albumin (Π_I_, eq. (3)), is washed down to 0, corresponding to a maximum limiting capillary pressure (P_C_^max^) described by:

(9) $$P_{C}^{\max}\;=\;\;\Pi_{P}\;+\;P_{A}$$

At the normal P_A _of 2 mmHg, this limiting capillary pressure is about 27 mm Hg. Since the intestinal capillary pressure is about 3 mm Hg greater than portal vein (P_PV_) pressure [15,23], this limit corresponds to a P_PV _of about 24 mm Hg. This is consistent with the experimental results of Witte et. al. [24] that the intestine did not leak large volumes of ascetic fluid until the portal pressure reached about 26 mm Hg (P_A _= 2 mmHg). Since P_C_^max ^depends on P_A _(eq. (9)), a patient with severe ascites and a P_A _of 15 mm Hg would not exceed this maximum until P_PV _was greater than 37 mm Hg, a value seldom observed clinically. (See Additional file 1, Section IE for more details).

#### B. Increase in liver sinusoidal pressure

Ascites readily forms when liver sinusoidal pressure is increased secondary to cirrhosis or obstruction of the hepatic veins or the inferior vena cava. This can be explained in terms of the model shown in Figure 1. As discussed above, the sinusoids are highly permeable to albumin and the liver tissue hydrostatic and colloid osmotic pressures are approximately equal to that of the sinusoids (eq. (7)), producing a hydrostatic pressure difference of (P_L _- P_A_) across the liver capsule. Increase in the sinusoidal pressure results in a flux of fluid into the interstitial space which elevates tissue pressure and hepatic lymph flow [17,18]. The crucial event in ascites is the rupturing of either the liver capsule and/or the surface lymphatics allowing high protein tissue fluid (Π_L _≈ Π_P_, eq. (7)) to leak into the peritoneal space.

This protein leak from the liver surface is supported by a number of direct observations. Experimental inferior vena cava constriction leads to obvious "weeping" of fluid droplets from the liver surface [25-27] while the other visceral surfaces appear dry [27]. Clinical evidence that the weeping liver is the source of ascites protein is provided by the observation of Dumont and Mulholland [28] that "Lymph leaking from clusters of bulging lymphatics on the liver capsule and at the porta hepatis often is encountered at laparotomy in patients with Laennec's cirrhosis". Kuntz and Kuntz [29] provide a dramatic image of "Numerous, partially ruptured lymphocysts ... on the liver surface with extravasation of protein-rich lymph in alcoholic cirrhosis" (Figure 16.5, p. 298). Tameda et. al. [30] observed "small lymphatic vesicles" on the liver surface in 65 out 372 cirrhotic subjects during peritoneoscopy. During laparoscopy, Heit et. al. [31] reported that 4 of 10 cirrhotic livers had surface "...lymphatic blebs indicating dilated lymphatic channels ... and all 4 of these cases were complicated by ascites." Blebs were not seen in the absences of ascites. (see Additional file 1, Section ID for more details).

The protein concentration in ascitic peritoneal fluid in portal hypertension is low, roughly equal to the protein concentration of intestinal lymph [13]. There is a definite "capillarization" of liver sinusoids in cirrhosis [32,33] and some authors [34,35] have suggested that the ascites protein is roughly equal to the low liver tissue protein produced by this capillarization. However, even in severe cirrhosis sinusoidal fenestra are still present [33] and the liver lymph protein/plasma ratio (which is a measure of tissue protein) is about 0.6, much greater than the corresponding ascites protein ratio of 0.17 [13]. Furthermore, this liver lymph protein/plasma ratio of 0.6 may underestimate the liver tissue protein because of lymphatic contributions from peribiliary capillaries and admixture from intestinal lymph [33]. An alternative explanation for this low ascites protein, which is directly supported by our quantitative model (Section III), is that the high protein fluid leaking from the liver pulls fluid osmotically from the "intestinal" tissue space (eq. (4)), diluting the peritoneal fluid protein until it roughly equals the intestinal tissue protein concentration. This implies that the rate of formation of ascitic fluid is determined primarily by two factors: the rate of protein leak from the liver and the interstitial protein concentration of the intestine, a concept alluded to in the older literature [36,37] but seldom discussed in recent reviews of ascites. This mechanism provides a straight forward explanation for use of the serum-ascites albumin gradient (SAAG) in the differential diagnosis of ascites (see Section IIE).

Since the ascites protein concentration in portal hypertension is only about 1/3 of that of the fluid leaking from the liver, the bulk of ascitic fluid is derived from the intestine rather than the liver. This fluid movement from the intestine into the peritoneum continues as long as the liver continues to leak protein. Homeostasis is achieved when the rising ascitic pressure (P_A_) increases the rate of peritoneal lymph and protein drainage until it equals the rate at which protein leaks from the liver. Although it has been hypothesized that increased ascites pressure (P_A_) should slow the rate of liver leak [17], as is discussed below, this should be a small to negligible effect because an increase in P_A _should produce a proportional increase in P_L_. The crucial parameter characterizing the rate of leakage of fluid from the liver is the value of (P_L _- P_A_). Experimental measurements of this parameter are discussed in the next section.

#### C. Interpretation of experimental hepatic "wedge" and "free" pressure and assessment of the pressure difference across the liver capsule (P_L _- P_A_)

Since sinusoids are leaky to protein, the liver hydrostatic tissue pressure (P_L_) is nearly equal to the "average" sinusoidal pressure (P_S_, eq. (7)). The site of the liver flow resistance is controversial, with evidence for both pre-sinusoidal [38] and post-sinusoidal [39] sites. Recent results suggest that the resistance is primarily in the sinusoids and is distributed relatively uniformly along the sinusoidal length both in normal and cirrhotic conditions [40-42]. Using this latter assumption:

(10) $$P_{S}\;\approx \;\;\left(P_{HV}\;+\;P_{PV}\right)\;/2\;\;\;\approx \;\;P_{L}$$

Since direct access to the portal vein requires a laparotomy, virtually all measurements have been obtained indirectly via hepatic vein catheterization. When flow is obstructed via "wedging" of a catheter in a hepatic vein, the measured pressure should equal the portal pressure (assuming no anastomoses between the vessels of the wedged liver segment and vessels with low pressure). Such is the case in cirrhosis, where "wedged" pressure measurements have been shown to equal portal vein pressures [43-46]. The pressure when the catheter is withdrawn to an unwedged ("free") position in a hepatic vein provides a measure of the hepatic vein (or inferior venal cava) pressure [47,48].

(11) $$P_{Wedge}\;\approx \;P_{PV}\;P_{Free}\;\approx \;P_{HV}$$

It is useful to briefly summarize the relations between all the pressures in the portal system (see Figure 1) for the normal condition:

(12) $$\begin{array}{c} Normal\;Relations:\;P_{A}\;<\;P_{RA}\;+2\\ P_{HV}\;=\;P_{RA}\;+2\;\;\;\;P_{PV}\;=\;P_{RA}\;+\;F_{L}R_{L}\;+2\;\;\;P_{C}\;=\;P_{RA}\;+\;F_{L}R_{L}\;\;+\;5\\ P_{S}\;\approx \;\left(P_{HV}+P_{PV}\right)/2\;\;\;\;\;\;\;\;\;\;\;P_{L}\;\approx \;P_{S}\;\;\;\;\;\;\;\;\;P_{I}\;\approx \;P_{A} \end{array}$$

where F_L _and R_L _are the liver flow and resistance and where the hepatic vein pressure (P_HV_) is assumed to be about 2 mm greater than the right atrial pressure (P_RA_) ([49] and see Table 1) and the intestinal capillary pressure (P_C_) is about 3 mm greater than the portal vein pressure (P_PV_) [15,50]. As these relations show, all the pressures in the portal system can be directly related to systemic right atrial pressure and the liver flow and resistance.

**Table 1:** Hemodynamic responses to drugs in cirrhotic patients

Drug	Δt	CO		HBF		P_RA_		Wedge		Free		P_HVPG_		Resistance		Δ(P_L _- P_A_)		Comments
	**Hours**	**Pre**	**Post**	**Pre**	**Post**	**Pre**	**Post**	**Pre**	**Post**	**Pre**	**Post**	**Pre**	**Post**	**Pre**	**Post**	**Eq. 16**	**Eq. 17**	

Spironolactone																		

[51]	1440	8.6	7.3	1.33	1.24	3.5	1.8	23.9	20.8	6.4	5.5	17.6	15.3	13.2	12.3	2.0	1.15	No ascites

[52]	168	6.75	NR	NR		NR		26.3	23.5	9.8	10.6	16.5	12.9	NR	NR	1.0	1.8	70% ascites

[53]	672	6.48	5.76	1.00	1.00	NR		18.7	15.7	2.2	2.8	16.5	12.9	16.5	12.9	1.2	1.8	No ascites

[54]	1344	5.5	4.9	1.0	0.94	NR		22.4	20.1	4.9	4.4	17.5	15.7	17.5	16.7	1.4	0.9	No ascites, unrestricted Na^+^

Furosemide																		

[53]	672	8.4	6.78	1.29	1.08	NR		17.6	16.7	3.9	3.1	13.7	13.6	10.6	12.5	0.85	0.05	No ascites

[55]	1	6.6	5.5	1.49	0.82	5	3	31.1	27.7	9.0	8.2	22.1	19.5	14.8	23.8	2.1	1.3	Acute affect, 60% ascites

Propranolol																		

[56]	1	7.6	5.9	1.06	0.862	3.7	5.5	23.8	22.9	5.7	7.0	18.1	15.9	17.1	18.4	-0.2	1.1	Varices and/or ascites

[57]	240	8.13	5.36	1.5	1.8	NR		29.7	26.9	8.6	10.1	21.1	16.8	14.1	9.3	0.65	2.15	45% ascites

[58]	672	6.05	5.0	0.93	0.826	1.9	1.1	19.3	14.5	3.6	3.2	15.1	11.4	16.2	13.8	2.6	1.85	No ascites

[59]	1/4	6.33	5.01	NR		NR		27.9	25.7	7.0	8.0	20.9	17.7	NR	NR	0.6	1.6	Mild--6% ascites

[59]	1/4	7.17	5.76	NR		NR		31.3	29.2	7.1	8.4	24.2	20.8	NR	NR	0.4	1.7	Moderate--72% ascites

[59]	1/4	9.03	5.92	NR		NR		32.1	29.6	8.8	9.5	23.4	20.1	NR	NR	0.9	1.65	Severe--100% ascites

[60]	1848	7.6	5.8	1.25	1.01	5.8	6.4	28.8	25.9	8.5	8.3	20.3	17.6	16.2	17.4	1.55	1.35	Varices, P_HVPG_> 12

[61]	2160	NR		NR		NR		27.5	26.0	10.0	13.9	17.3	12.1	NR	NR	-1.2	2.6	100% variceal bleed

[62]	336	NR		NR		8.8	12.2	34.5	32.7	15.8	17.1	18.7	15.45	NR	NR	0.25	1.63	75% ascites

Timolol																		

[63]	1	NR		NR		NR		24.9	23.6	8.4	10.0	16.5	14.4	NR	NR	-0.15	1.05	100% varices

Carvedilol																		

[60]	1848	7.5	6.4	1.39	1.28	4.6	5.5	26.4	23.5	7.3	8.2	19.0	15.2	13.7	11.9	1.0	1.9	Varices, P_HVPG_> 12

Clonidine																		

[64]	168	6.41	5.40	1.05	1.33	5.2	3.4	30.4	27.4	10.2	9.7	20.1	17.6	19.1	13.2	1.75	1.25	100% Ascites

[65]	3/4	6.40	5.36	0.96	1.22	NR		31.3	28	11.0	11.4	20.3	16.6	21.1	13.6	1.45	1.85	100% Ascites

[66]	448	7.8	6.7	1.2	1.1	2.6	4.2	23.9	24.6	5.1	8.7	18.8	15.9	15.7	14.4	-2.15	1.45	40% ascites

Nitroglycerin																		

[67]	≈1/10	NR		NR		NR		22.5	18.9	4.6	3.6	17.9	15.1	NR	NR	2.3	1/4	Sublingual

[68]	1	NR		0.93	0.919	NR		26.0	22.1	8.4	8.6	17.6	13.6	NR	NR	1.85	2	Transdermal

[69]	1/3	7.42	6.75	1.34	1.1	6.1	4.0	29.2	29.3	8.3	8.2	20.9	21.1	15.7	14.4	0	00.1	Low dose IV, 53% ascites

Vasopressin																		

[70]	1/2	NR		NR		NR		28.6	24.0	5.7	6.3	22.9	17.7	NR	NR	2.0	2.6	Bleeding varices, 40% ascites

[71]	1/2	7.6	6.5	1.89	1.06	5.0	7.5	26.6	23.6	7.6	9.1	19.0	14.6	10.1	13.8	0.75	2.2	30 min IV inf

[72]	1/2	7.14	5.77	1.35	0.91	NR		17.8	12.7	6.7	7.3	11.1	5.4	8.22	5.93	2.25	2.85	40% ascites, IV inf

Terlipressin																		

[73]	1	NR	NR	NR	NR	5.2	9	25.4	25.0	6.0	8.1	19.4	16.8	NR	NR	-0.85	1.3	Variceal gradient reduction

Captopril																		

[74]	504	NR		NR		NR		22.9	20.7	15.0	12.1	7.58	9.32	NR	NR	2.55	-0.5	100% ascites

[61]	2160	NR		NR		NR		24.8	21.1	9.3	7.1	15.6	13.0	NR	NR	2.95	1.3	100% variceal bleed

Enalaprilat																		

[75]	1/2	6.7	6.9	NR		NR		32.1	25.6	11.2	10.8	21.0	16.1	NR	NR	3.45	2.45	IV infusion

Losartan																		

[62]	336	NR		NR		8.4	8.55	32.4	28.31	13.21	14.26	19.21	14.15	NR	NR	1.53	2.53	63% ascites

[76]		5.8	5.7	NR		NR		20.3	17.3	4.9	3.7	15.4	13.6	NR	NR	2.1	0.9	Pre-ascitic, 4 week

Saralasin																		

[77]	≈1/2	6.5	5.97	1.42	1.35	4.8	4.3	28	24.0	10.8	10.2	17.2	13.78	12.1	10.2	2.31	1.71	100% ascites, IV inf

Somatostatin																		

[72]	1/2	6.59	6.38	3.7	1.1	0.89	4.1	19.5	16.2	7.2	7.6	12.3	8.6	3.32	7.82	1.45	1.85	40% ascites, IV inf

Fenoldopam																		

[78]	1	8.9	10.5	1.5	1.6	6.1	4.4	24.6	28.0	9.2	8.7	15.4	19.3	10.23	12.1	-1.45	-1.95	MAP: 84.6-61.8 decrease

Δt = time interval pre--post (hours), CO = cardiac output (liters/min), HBF = hepatic blood flow (liters/min), P_RA _= right atrial pressure (mm Hg),

Wedge = portal wedge pressure (mm Hg), Free = free portal pressure (mm Hg), P_HVPG _= Wedge - Free, Resistance = hepatic flow resistance = Gradient/HBF (mm Hg/liter),

Δ(P_L _- P_A_) = change in pressure difference across the liver capsule--(pre - post), MAP = mean arterial pressure

There is a dramatic change in these relations if there is gross ascites and the ascites pressure (P_A_) becomes greater than the normal hepatic vein pressure. Since the hepatic veins (and inferior vena cava) are collapsible, the hepatic vein pressure must be minimally greater than the intra-abdominal pressure, so that P_A _≈ P_HV _≈ P_Free_. Hernriksen et. al. [47] have directly confirmed this relation and shown that P_Free _≈ P_A _over a wide range of pressures in patients with ascites. This condition produces a different set of relations:

(13) $$\begin{array}{c} Ascites:\;\;P_{A}\;>\;=P_{RA}\;+2\\ P_{HV}\;=\;P_{A}\;\;\;\;\;\;\;\;\;\;\;\;\;P_{PV}\;=\;P_{A}\;+\;F_{L}R_{L}\;\;\;\;\;\;\;\;\;P_{C}\;=\;P_{A}\;+\;F_{L}R_{L}\;\;+\;3\\ P_{S}\;\approx \;\left(P_{HV}+P_{PV}\right)/2\;\;\;\;\;\;\;\;\;\;\;P_{L}\;\approx \;P_{S}\;\;\;\;\;\;\;\;\;P_{I}\;\approx \;P_{A} \end{array}$$

As can be seen, for this condition the portal pressures become linearly dependent on P_A _and are uncoupled from the systemic right atrial pressure. This shift between these two pressure domains, which commonly is ignored, has important implications for interpreting portal pressure measurements (see below). At high peritoneal pressure, all the liver and intestinal pressures become additively dependent on P_A _and changes in P_A _have no effect on the relative pressure relations. This has already been referred to above (Section IIB) when it was concluded that increases in ascitic pressure do not change the value of (P_L _- P_A_) and therefore do not slow the leak of liver protein. This constancy of (P_L _- P_A_) as P_A _increases is based on the assumption that the liver flow (F_L_) and resistance (R_L_) are constant (eq. (13)). In fact, one might expect a small decrease in F_L _as increases in P_A _and P_PV _leads to increased portal to systemic shunting. This decrease in F_L _should slightly decrease the value of (P_L _- P_A_). This prediction is directly supported by the measurements of Luca et. al. [79] of the free and wedge pressure before and after paracentesis. The wedge pressure fell by 7.4 mm Hg (31.9-24.5) and there was a nearly equivalent 5.9 mm Hg fall in the free pressure with P_L _- P_A _(eq. (15)) decreasing by only 0.85 mm Hg.

The difference between the wedge and free pressure, the "hepatic vein pressure gradient" (P_HVPG _= P_Wedge _- P_Free _≈ P_PV _- P_HV _= F_L_R_L_), is directly proportional to the resistance of the liver to portal flow. Thus, this gradient commonly is employed to characterize the severity of portal hypertension in cirrhotic subjects. P_HVPG _is also used clinically as a surrogate measure of the driving pressure for the formation of ascites. This can be misleading because it ignores the importance of the two pressure domains (eqs. (12) and (13)) discussed above. The critical parameter characterizing the rate of ascites formation is the pressure difference across the liver capsule (P_L _- P_A_). With low intraperitoneal pressure, this pressure difference (calculated using eq. (12)) equals:

(14) $$\begin{array}{c} P_{L}\;-\;P_{A}\;\;=\;\left(P_{PV}\;+\;P_{HV}\right)/2\;\;-\;\;P_{A}\\ \;\;\;\;\;\;\;\;\;\;\;\;\;\;\approx \;\left(P_{Wedge}\;+\;P_{Free}\right)/2\;\;-\;P_{A\;\;}\;\;\;\;\; \end{array}$$

For the case of high intraperitoneal pressure (that occurs with appreciable ascites), P_HV _= P_A _≈ P_Free _(eq. (13)) and (P_L _- P_A_) is described by:

(15) $$\begin{array}{c} P_{L}\;-\;P_{A}\;\;=\;\;\left(P_{PV}\;+\;P_{HV}\right)/2\;\;-\;P_{A}\;\;\;=\;\;\left(P_{PV}\;+\;P_{A}\right)/2\;-\;P_{A}\\ \;\;\;\;\;\;\;\;\;\;\;\;\;\;\approx \;\;\left(P_{Wedge}\;-\;P_{Free}\right)/2\;\;=\;P_{HVPG}/2 \end{array}$$

It is only for this latter case that (P_L _- P_A_) is directly determined by P_HVPG_. In the absence of gross ascites, determination of (P_L _- P_A_) requires an estimate of ascites pressure (which usually is neglected in most studies of portal hemodynamics). Recognition of the importance of the two pressure domains and correctly calculating (P_L _- P_A_) is crucial since, as will be shown, minor differences (several mm Hg) in pressure may determine the presence or absence of ascites. The discussion of the action of β-blockers (Section IIF) provides an illustration of the importance of the difference between calculations based on eq. 14 versus eq. 15.

In subjects with normal livers, P_Wedge _and P_Free _are about 6 and 4 mm Hg, respectively [80-82]. Since direct measurement of P_A _in normal subjects with no overt ascites is difficult, indirect approaches have been employed [83]. Using the urinary bladder measurements, Cobb et. al. [14] reported values of about 2 mm Hg for P_A _in the normal supine subject. Substituting this value into eq. (14), yields a (P_L _- P_A_) of about 3 mm Hg for normal subjects. Estimates of the value of (P_L _- P_A_) in ascites subjects is uncertain because, as discussed above, investigators have failed to distinguish between the two pressure domains and direct measurements of P_A _are not available. There is a weak correlation between P_HVPG _and the presence or absence of ascites. In 3 studies of P_HVPG _in cirrhotic patients, the average values in subjects without versus with ascites were 11.5 vs 16 [84]; 17.7 vs 19.1 [85] and 14.9 vs 17.8 mm Hg [86]. In a prospective follow up of 122 cirrhotics undergoing a TIPS procedure, Casado et. al. [87] reported that ascites was a problem only in patients whose P_HVPG _remained greater than 12 mm Hg. Substituting this value into eq. (15), indicates that the value of P_L _- P_A _must be at least 6 mm Hg (twice the normal value) before appreciable ascites is formed. This observation supports the assumption in our model that there is a critical value of P_L _- P_A _above which the liver capsule and/or lymphatics rupture, allowing leakage of high protein tissue fluid into the peritoneal space.

#### D. Intra-abdominal lymph drainage (J_lymph_)

The volume of ascites reaches a steady-state when the rate of leakage of protein from the liver equals the rate of protein removal by lymph. Most of the peritoneal cavity is drained via diaphragmatic lymphatics that drain into the right lymph duct. These lymphatics have complex stomatal openings on the peritoneal surface that provide fixed drainage sites [19]. These drainage sites are like holes in a balloon and, unlike the hepatic vein or inferior vena cava, cannot be collapsed by high intraperitoneal pressure. Since it is not possible to directly measure this lymph flow, indirect methods have been employed. The standard approach is to measure the kinetics of removal of labeled albumin from peritoneal fluid. A major area of controversy in the peritoneal dialysis literature is the interpretation of these measurements since the rate of disappearance of label from peritoneal fluid seemingly is about 10 times greater than the rate of appearance of this label in plasma [88,89] due to sequestration of the albumin in tissue. The weight of evidence suggests that lymph flow should be determined from the rate of appearance of the peritoneal label in plasma. This rate (J_lymph_) is only about 10 ml/hour when 2 liters of fluid is instilled during peritoneal dialysis [90,91]. Henriksen et. al. [92,93] have shown that in patients with cirrhotic ascites, J_lymph _is much greater, about 50-60 ml/hour. The factors responsible for this increased lymph flow are not well understood. These patients had greater pressures (P_Free _of about 12 mm Hg) and ascitic volumes (about 7 liters) than the dialysis patients which may account for the difference. Zink and Greenway [94] have shown in cats that peritoneal fluid and protein absorption had an approximately linear dependence on pressure.

It is well established that cirrhotic patients can be in a steady state with roughly constant ascites volumes for long time periods. This implies that as ascites volume increases, there must be some compensating effect that produces a balance between the rate of protein leak and removal. As discussed above, a rise in P_A _should not have a marked effect on the protein leak rate determined by (P_L _- P_A_) since this increase in P_A _produces an equivalent increase in P_L_. Thus, the homeostatic mechanism presumably is that an increase in P_A _increases J_lymph _as has been experimentally observed in cats [94]. Quantitative support for this in humans is provided by the measurements of Shear et. al. [95] of the rate of formation of ascites following paracentesis. If one assumes that the rate of lymph flow was 60 ml/hour pre-paracentesis (see above) and 10 ml/hour post-paracentesis (as observed in dialysis patients), the predicted net rate of ascites formation immediately after paracentesis would be about 50 ml/hour (1.2 liters/day), which is similar to what was observed by Shear et. al. [95]. However, this is indirect evidence and, although lymph flow must balance protein leak at steady state, the exact rate of this flow in various situations needs further study.

#### E. Protein concentration in ascitic fluid and the serum-ascites albumin gradient (SAAG)

The model predicts that 1) hepatic lymph protein should be approximately equal to plasma protein (eq. (7)); 2) as the liver sinusoidal pressure is raised, a concomitant rise in intestinal capillary pressure should wash down the protein concentration in the intestinal tissue; and 3) ascitic fluid protein should approximately equilibrate with this washed down intestinal tissue protein (eq. (8)) (which equals intestinal lymph protein). A corollary of predictions 2 and 3 is that as the severity of portal hypertension increases, which causes an increasing intestinal capillary pressure and decreasing intestinal tissue protein, the ascitic fluid protein concentration should also fall. Witte et. al. [13] measured the protein concentration in ascites fluid and hepatic and intestinal lymph in patients in varying stages of ascites. The measured lymph protein/plasma total protein ratios were: control: hepatic = 0.88; intestinal = 0.7; early stage cirrhosis: hepatic = 0.8, intestine = 0.8, ascites = 0.65; late stage: hepatic = 0.6; intestine = 0.12; ascites = 0.17. These experimental observations are in qualitative agreement with the above predictions. An important differential diagnostic marker of ascites is the value of the serum-ascites albumin gradient (SAAG). A SAAG of ≥1.1 gm% is strongly indicative of portal hypertension (either cirrhotic or cardiac ascites) while all other causes of ascites (e.g. infections, malignancy) tend to have smaller gradients [96]. While the diagnostic accuracy of the SAAG has been demonstrated empirically, the physiology underlying this measurement has received little attention. The values of the SAAG in various conditions follow directly from the assumption in the model that intestinal tissue and ascites fluid have similar albumin concentrations (eq. (8)). In subjects with normal portal pressure these concentrations are high, about 70% of the plasma value, resulting in a low SAAG. In contrast, the increase in intestinal capillary hydrostatic pressure and the accompanying wash down of intestinal interstitial protein produced by portal hypertension of any etiology results in low ascites protein concentrations, hence a high SAAG.

Although the above discussion is focused primarily on cirrhotic ascites, cardiac ascites is also associated with a SAAG of ≥1.1 gm%. In a series of patients with chronic hepatopathy (68% with ascites), the average value of the free hepatic vein pressure was 17 mm Hg, with a P_HVPG _of 2 mm Hg [97]. Thus, the liver sinusoidal pressure is about 18 mm Hg which, presumably, results in rupture of liver lymphatics and leakage of protein into the peritoneal space. The corresponding intestinal capillary pressure is about 22 mm Hg (3 mm Hg greater than portal vein pressure) which should wash down intestinal tissue albumin to a low value. Osmotic equilibration of the ascitic albumin with this intestinal tissue results in a large SAAG.

#### F. Medical treatment of ascites

Given the widespread use of diuretics to treat ascites, it is surprising that the mechanism of benefit of these drugs has received little attention. The challenge is relating the systemic effects of diuretics (alterations in blood pressure, blood volume, systemic resistance and capacitance, etc.) to the two local primary factors that determine ascites volume in the above model: 1) protein leak from the liver surface as a result of increased (P_L _- P_A_) and 2) intra-abdominal lymph drainage. In this section we will compare the hemodynamic actions of diuretics that alleviate ascites, with the actions of a number of other drugs that have been evaluated for potential utility in the treatment of portal hypertension

Table 1 summarizes the reported hemodynamic changes produced by 15 different drugs in cirrhotic subjects. As discussed above (Section IIC), there are two different relationships for (P_L _- P_A_), depending on whether there is no ascites and the intra-abdominal pressure (P_A_) is assumed to be small (eq. (14)) or there is appreciable ascites with an increased P_A _(eq. (15)). The penultimate column in Table 1 lists the calculated drug-induced changes in (P_L _- P_A_) for both of these cases using the following equations:

(16) $$\Delta \left(P_{L}\;-\;P_{A}\right)\;\;=\;\;\;\;\left(P_{Hep\;Wedge}^{pre}\;+\;P_{HepFree}^{pre}\;-\;P_{Hep\;Wedge}^{post}\;-\;P_{HepFree}^{post}\right)/2\;\;\;\;\;\;\;\;\;\;\;\;No\;ascites$$

(17) $$\Delta \left(P_{L}\;-\;P_{A}\right)=\;\;\left(P_{HPVG}^{pre}\;-\;P_{HPVG}^{post}\right)/2\;\;\;\;\;\;\;\;\;\;\;\;\;\;\;\;\;\;\;\;\;\;\;\;\;\;Ascites$$

where it is assumed for eq. (16) that the drug does not change the intra-abdominal pressure. For most of the studies listed in Table 1 there was either no or mild ascites, and eq. (16) should be used. Also listed in Table 1 is the change in the hepatic blood flow (F_L_), the gradient (P_HVPG_), the corresponding liver resistance (R_L _= P_HVPG_/F_L_) and the right atrial pressure (P_RA_).

The administration of the spironolactone or furosemide decreased P_HVPG _by about 20% [51-55] (Table 1). This decrease primarily resulted from a decrease in the wedge pressure whereas there was only a small change in free hepatic pressure. This decrease in P_HVPG _was roughly proportional to the decreased hepatic blood flow; hence there was little change in hepatic resistance. It is also notable that, in the two studies where right atrial pressure was measured, the diuretics produced about a 2 mm Hg fall in pressure. In summary, the alterations in local peritoneal fluid balance induced by diuretic therapy presumably are the result of two major systemic changes: 1) a decrease in cardiac output with a corresponding fall in hepatic blood flow and P_HVPG_, and 2) a decrease in right atrial pressure.

Administration of non-selective beta blockers (propranolol, timolol, carvediol), cause reductions in P_HVPG _comparable to that observed with diuretics (Table 1). However, beta blockers are not considered useful for the treatment of ascites, and Rector and Reynolds [98] have shown that the addition of propranolol to diuretic therapy reduces renal sodium excretion and may interfere with the action of diuretics. On the other hand, beta-blockers consistently have been demonstrated to reduce the incidence of bleeding from esophageal varices whereas no such benefit has been noted with diuretics. Surprisingly, there has been little discussion of this paradoxical difference in efficacy of diuretics versus beta-blockers in the treatment of complications of portal hypertension, given that both classes of drugs seemingly produce similar reductions in portal hemodynamics as measured by the P_HVPG_. An important difference between the action of these drugs is that in marked contrast to diuretics, about 50% of the decrease in P_HVPG _induced with beta blockers results from an increase in the free hepatic pressure (Table 1). Thus the estimated decrease in (P_L _- P_A_) using eq. (16) is less than half that observed with diuretics. Again, the local changes can be directly associated with the systemic effects, with the decrease in the gradient explained by the decrease in cardiac output and hepatic blood flow (with negligible change in resistance) and the increase in the free portal pressure explained by the increased right atrial pressure. These data demonstrate why P_HVPG _is not directly correlated with (P_L _- P_A_) in patients without gross ascites. For example, a drug that increased the free hepatic pressure but had no effect on the wedge pressure would decrease P_HVPG _but would actually increase (P_L _- P_A_) according to eq. (16). It has been shown that the development of varices or variceal hemorrhage is strongly correlated with both the absolute value of P_HVPG _[99] and the response of P_HVPG _to beta blockers [48]. For varices, the important parameter is the difference between the portal vein pressure and the pressure in the shunted systemic vein. One would expect that P_HVPG _should be a good approximation of this pressure difference. Thus, P_HVPG _may influence the tendency to bleeding from varices, even though this pressure gradient does not directly correlate with (P_L _- P_A_), which is the crucial parameter for the development of ascites.

Clonidine, an α_2 _receptor agonist, is unique among the 15 drugs in Table 1 in that its major action seems to be a decrease in hepatic resistance which produces a corresponding decrease in P_HVPG _with little change in hepatic blood flow [64-66]. In clinical trials of clonidine for the treatment of ascites, clonidine by itself was not effective but the combination of clonidine and spironolactone produced a greater weight loss and ascites decrease than spironolactone alone [100]. Because clonidine does not decrease portal blood flow, it is not regarded as an effective treatment for gastroesophageal varices [101,102]

Also listed in Table 1 are the portal hemodynamic responses to nine other drugs. The activation of the renin-angiotensin-aldosterone (RAAS) system has a central role in the standard "forward theory" of ascites formation [6]. The RAAS antagonists in Table 1 (captopril, enalapril, losartin, saralasin) reduce P_HVPG _with little or no change in P_Free_. A recent systematic meta-analysis of the RAAS antagonists concluded that the P_HVPG _response was a function of the Child Pugh class [103]. Despite this decrease in P_HVPG_, ACE inhibitors do not seem to be of benefit in the treatment of ascites [104]. Although vasopressin and terlipressin (vasopressin agonist) decreased P_HVPG_, this decrease resulted primarily from an increase in P_RA _and P_Free _(Table 1) and, therefore, they are unlikely to be beneficial in the treatment of ascites. The most recent candidate drug classes for the treatment of ascites are the vasopressin V2 receptor antagonists (vaptans). These drugs increase plasma sodium by increasing solute free water excretion and it was hoped they would be useful in ascites. Although short term trials of satavaptan suggested a positive effect, a recent long term randomized trial concluded that it is not beneficial in the treatment of ascites [105]. It is clear from the results in Table 1 that one cannot explain the therapeutic action of diuretics based only on the reduction of P_HVPG _since many other drugs that apparently are not effective in the treatment of ascites have similar actions on P_HVPG._

Serum albumin concentration commonly is decreased in cirrhosis. The resultant fall in plasma oncotic pressure is only partially offset by the increased in serum globulins observed in cirrhosis. The use of albumin infusion for the treatment of ascites is controversial. In a comprehensive review of this literature, Trotter et. al. [106] concluded that, although short term therapy (11 to 16 days) was not useful, there was suggestive evidence that long duration albumin infusion (27 days to 2 years) in association with diuretics was beneficial in terms of ascites resolution and ascites redevelopment. In the only controlled trial of albumin in the treatment of ascites, Gentilini et. al. [107] found that addition of albumin to diuretic therapy resulted in a statistically significant increase in the number of responders and significantly reduced the redevelopment of ascites. The interpretation of these results is complicated because albumin also has systemic renal and cardiovascular effects. For example, albumin infusions may increase blood volume and central venous pressure which would increase (P_L _- P_A_) and exacerbate the ascites. The effects of albumin predicted by our model are small because any change in the amount of protein leaking from the liver will be roughly balanced by the change in the intestinal tissue protein. The model predicts that albumin would affect ascites formation at very high portal pressures because, as was discussed in Section IIA (see eq. (9)), there is a limit to the intestinal capillary pressure at which Starling force balance can be achieved and this limit is set by the plasma colloid osmotic pressure. Thus, in severe portal hypertension that produces intestinal capillary pressures that approach or exceed plasma oncotic pressure, there should be an increase in the rate of ascites formation with decreasing albumin. The quantitative model discussed in Section III provides predictions of the influence of plasma oncotic pressure on ascites volume over the entire range of portal pressures.

If one assumes that the model presented in this review is correct, then diuretics must act through the two local factors (P_L _- P_A_) and J_lymph_. Diuretics decrease (P_L _- P_A_) from 1 to 2 mm Hg, which represents about a 10-20% decrease from the initial value. Another action of diuretics which stands out from all the other drugs listed in Table 1 is the relatively large decrease in right atrial pressure (P_RA_). This lowering of P_RA _by spironolactone or furosemide is apparently not dependent on the diuretic action since decreases of 2-5 mm Hg have been observed in chronic renal failure patients on hemodialysis [108,109]. This decrease in P_RA_, which has not been previous emphasized, has two separate effects on ascites. First, for low peritoneal pressures (eq. (12)), P_L _is additive to P_RA _and a decrease in P_RA _directly reduces (P_L _- P_A_) and the liver leak rate. Second, as discussed in more detail in the description of the quantitative model (Section III), this decrease in P_RA _may increase the rate of peritoneal fluid drainage (J_lymph_). Although the effect of diuretics on protein leak and J_lymph _are relatively small, if, pre-diuretic, the rate of leak and lymph flow are equal and opposite, these small changes can shift the balance and lead to a reduced ascitic volume. In the next section a quantitative model is developed that demonstrates that, under some conditions, these small changes (20% reduction in P_HVPG _and 3 mm Hg reduction in P_RA_) surprisingly can lead to the complete reabsorption of ascites. We believe that this is the first attempt to provide a mechanistic explanation for the efficacy of diuretics.

### III. Quantitative physical modeling

This section will present a quantitative model of ascites formation and removal and the influence of various physiological perturbations on the model's predictions. The first part of this analysis assumes that the system is in a steady state with a constant ascites volume. For this case J_I _(eq. (4)) equals J_C _(eq. (1)) and (assuming P_I _= P_A_) one can express the fluid flux from the blood to the intraperitoneal space as:

(18) $$\begin{array}{c} J_{I}\;=\;\;L_{T}\left[\left(P_{C}\;-\;P_{A}\right)\;-\;\left(\Pi_{P}\;-\;\Pi_{A}\right)\right]\\ L_{T}\;=\;L_{I}L_{C}/\left(L_{I}\;+\;L_{C}\right) \end{array}$$

where L_T _is the total blood to peritoneal space hydraulic conductance of the intestine (i.e., the series conductance of the capillary and mesenteric membranes). The model further assumes that the intact liver capsule is impermeable to protein, and fluid flux from the liver into the peritoneal cavity occurs only when a critical pressure difference (= P_Break_) between liver tissue (P_L_) and peritoneal space (P_A_) is reached that results in rupture of the liver lymphatics and/or capsule. Above this break pressure, the rate of flux of fluid from the liver lymphatics into the peritoneal cavity is a function of the difference between the liver tissue and intraperitoneal pressure (for pressure differences greater than P_Break_). Thus:

(19) $$\begin{array}{c} J_{L}\;=\;\;L_{L}\;\left(P_{L}\;-\;P_{A}\;-\;P_{Break}\right)\;\;\;\;P_{L}\;\;-\;P_{A}\;>\;P_{Break}\\ \;\;=\;\;0\;\;\;\;\;\;\;\;\;\;\;\;\;\;\;\;P_{L}\;\;-\;P_{A}\;<=\;P_{Break} \end{array}$$

where L_L _is the conductance of the ruptured lymphatics.

The model assumes that lymph flow from the peritoneal cavity is proportional to the difference between the intraperitoneal (P_A_) and right atrial pressures (P_RA_):

(20) $$\begin{array}{c} J_{Lymph}\;=\;L_{Y}\left(\;P_{A}\;\;-\;P_{RA}+\;P_{\min}\right)\;\;\;\;P_{A}\;>\;P_{RA}\;-\;P_{\min}\\ \;\;\;\;\;\;\;\;\;=\;\;0\;\;\;\;\;\;\;\;\;\;\;\;P_{A}\;\;\;<\;P_{RA}\;\;-\;P_{\min} \end{array}$$

where P_min _determines the zero flow rate and will be assumed equal to 2 mm Hg. The steady state ascites volume requires that the intra-abdominal lymph flow equals the sum of the intestinal plus liver fluxes:

(21) $$J_{lymph}\;=\;J_{I}\;+\;J_{L}$$

Also, the total protein leak from the liver must equal the amount removed by the lymph:

(22) $$\;m\;\Pi_{P}\;J_{L}\;\;=\;\;\Pi_{A}\;J_{lymph}$$

where m is the concentration of protein in the liver exudate as a fraction of the plasma protein (Π_P_). It will be assumed that the intestinal capillary pressure (P_C_) is 3 mm Hg greater than the portal vein pressure (P_P_) and that the hepatic vein pressure (P_HV_) is either a) 2 mm greater than the right atrial pressure (P_RA_) in the absence of gross ascites; or b) equal P_A _if P_A _is greater than (P_RA _+2):

(23) $$\begin{array}{c} P_{C}\;=P_{RA}\;+\;P_{HPVG}\;+\;5\;\;\;\;\;\;\;\;\;\;\;\;\;\;\;\;\;\;\;\;\;\;\;\;\;\;\;\;\;P_{A}\;<\;P_{RA}\;+2\\ \;\;\;\;=\;P_{A}\;+\;P_{HPVG}\;+3\;\;\;\;\;\;\;\;\;\;\;\;\;\;\;\;\;\;\;\;\;\;\;\;\;\;\;\;\;\;P_{A}>P_{RA}\;+\;2 \end{array}$$

where the gradient P_HVPG _is the pressure drop across the liver (= P_PV _- P_HV_). Finally, it will be assumed that the ascites volume is proportional to the ascites pressure:

(24) $$Volume\;\;=\;V_{\min}\;+\;\;D*\left[P_{A}\;-\;P_{\min}\right]$$

where V_min _is the ascites volume found normally (100 ml) when P_A _= P_min _= 2 mm Hg and D is the peritoneal compliance. Equations (18)-(24) provide a complete steady state description of the system.

Estimates of the values of the above parameters can be obtained by using the following values for a "typical" ascites patient: P_HVPG _= 20 mm Hg, ascitic hydrostatic pressure (P_A_) = 10 mm Hg [47], ascites osmotic pressure (Π_A_) = 30% of plasma [47,96], an elevated P_RA _= 5 mm Hg (Table 1) and J_lymph _= 55 ml/hour [92,93]. It will be assumed that P_Break _= 8 mm Hg and that the liver exudate has a protein concentration equal to 0.8 (= m) of the plasma, similar to the value found for liver lymph [13]. In the following, the equivalent colloid osmotic pressure (Π) will be used for the protein concentration, assuming a plasma value (Π_p_) = 25 mm Hg. Using J_lymph _= 55 ml/hour in eq. (20), L_Y _= 7.86 ml/hour/mm Hg (assuming P_RA _= 5 and P_min _= 2). Diluting the liver exudate protein from a plasma fraction of 0.8 (= m) to the ascites value of 0.3 requires J_L _= 0.375J_Y _= 20.6 ml/hour and J_I _= 0.625J_Y _= 34.4 ml/hour. Using the assumed "typical" ascitic pressures (P_HV _= 10, P_P _= 30, P_L _= 20 and P_L _- P_A _= 10 mm Hg), the net driving force above P_Break _is 2 mm Hg, corresponding to an L_L _= 10.3 ml/hour/mm Hg (eq. (19)). Using these pressures and a Π_P _of 25 mm Hg in eq. (18), the value of the "intestinal" ultrafiltration coefficient = L_T _= 6.25 ml/hour/mm Hg. The peritoneal dialysis literature [110] uses a value for L_T _of 4.5 ml/hour/mm Hg for a 2 liter exchange volume. The exchange surface would be larger for the 5 to 10 liter ascitic volumes that are common in ascites patients. Thus, a somewhat higher L_T _(6.25 ml/hour/mmHg) was employed in the model. Finally, a compliance of the peritoneal cavity (D) of 0.8 liters/mm Hg will be used, based on experimental measurements in humans of the changes in peritoneal pressure following paracentesis [111,112].

**Figure 2:**
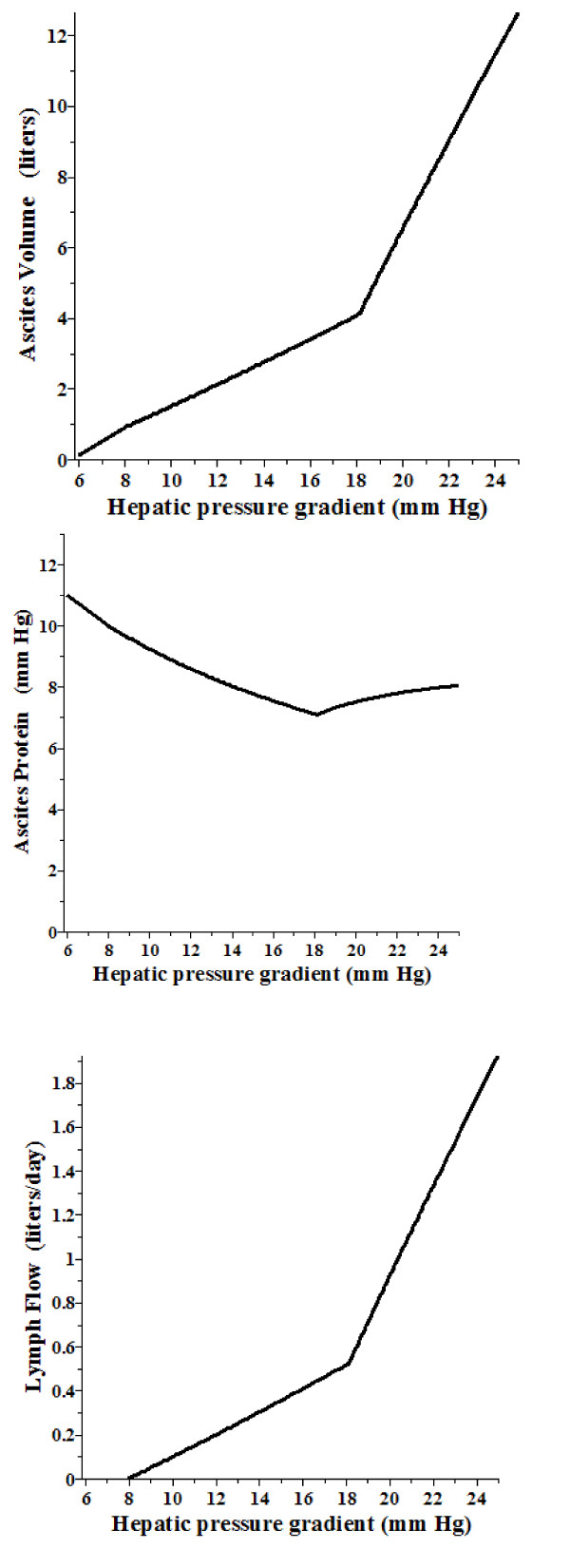
**The model prediction for the steady state ascites volume (top), colloid osmotic pressure (middle) and lymph flow (bottom) as a function of the hepatic vein pressure gradient (P_HVPG _= wedge - free)**.

Figure 2 shows the steady state peritoneal volume (top), protein concentration (middle) and lymph flow as the P_HVPG _varies from 6 mm Hg (the pressure when liver exudation starts) to 25 mm Hg. At a gradient of 18.15 mm Hg, there is shift between the low pressure domain where P_HV _= P_RA _+ 2, and the high ascitic pressure domain where P_HV _= P_A _(eq. (23)). The ascites protein concentration is expressed in terms of its equivalent colloid osmotic pressure. (For a normal subject with P_HVPG _= 2, P_A _= 2, P_RA _= 2, P_HV _= 4, the osmotic activity of the steady state ascites protein concentration should be about 20 mm Hg.) The ascites protein stays in a rather narrow range, falling from about 11 mm Hg when the ascites fluid begins to form, to a minimum of about 7 mm Hg at P_HVPG _of 18 mm Hg and then slowly rising to about 8 mm Hg. Assuming an albumin/total protein fraction of 0.65, these values correspond to albumin concentrations of about 2.5, 1.75 and 1.95 gm% respectively [113]. For the assumed plasma colloid osmotic pressure of 25 mm Hg (albumin concentration of 4.45 gm%), these values correspond to a SAAG of 1.95, 2.75 and 2.5 gm%. The fall in ascites protein results from the wash down of intestinal tissue protein as the capillary blood pressure increases. At high P_HVPG _the leak of high protein fluid from the liver makes a relatively greater contribution to the total ascitic fluid formation, producing the increase in the ascitic protein concentration.

**Figure 3:**
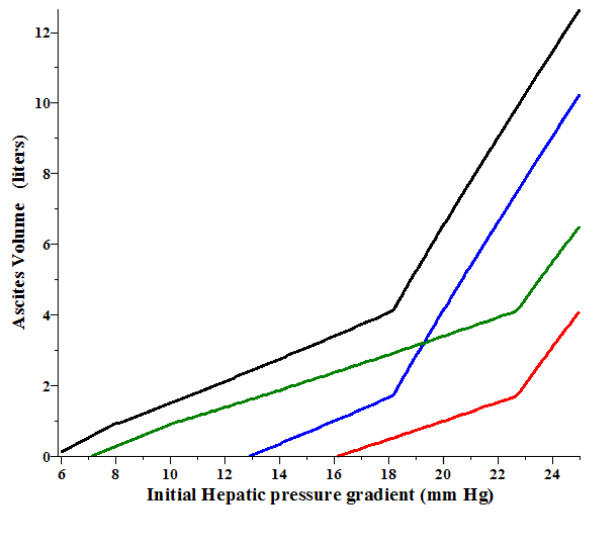
**Decrease in the ascites volume from the untreated case (black line) produced **by a) **decrease in P_HVPG _by 20% (green), **b) **decrease of right atrial pressure (P_RA_) from 5 to 2 mm Hg (blue) and **c) **decrease in both P_HVPG _by 20% decrease and P_RA _from 5 to 2 mm Hg (red) as a function of the original P_HVPG _before treatment**.

It is of interest to see how the ascites volume is altered by the hemodynamic changes produced by diuretics. As indicated in Table 1, diuretics reduce both the hepatic venous pressure gradient (P_HVPG_) and the right atrial pressure (P_RA_). Figure 3 shows how the steady state ascites volume in the original untreated condition (black line) is altered by either a) a 20% decrease in gradient (green line); b) a reduction in P_RA _from 5 to 2 mm Hg (blue line), or c) a combination of both (a) and (b) (red line) as a function of the initial P_HVPG_. For an initial gradient of 20 mm Hg, the ascites volume is reduced from about 6.5 to 1.0 liter if diuretics induced both of the above noted changes in P_HVPG _and P_RA_. If the initial gradient is 16 mm Hg or less, diuretics should produce complete reabsorption of the ascites. The reductions in P_HVPG _and P_RA _both contribute to the ascites reabsorption, but in different ways. At high initial P_HVPG _(> 18 mm Hg), when the hepatic vein pressure becomes decoupled from P_RA _(eq. (13)), lowering P_RA _(Figure 3, blue line) has a relatively small effect, while lowering P_HVPG _(green line) has a larger effect. In contrast, in the low pressure regime when the hepatic vein pressure is equal to (P_RA _+ 2) (eq. (12)) lowering P_RA _(blue line) has a dramatic effect. For example, if the initial P_HVPG _is less than 13 mm Hg, lowering P_RA _from 5 to 2 mm Hg is enough by itself to completely resolve the ascites. These predicted results indicate that the small changes in P_HVPG _and P_RA _clinically achievable with diuretics (see Table 1) can link the systemic changes induced by diuretics to fluid accumulation in the abdomen.

**Figure 4:**
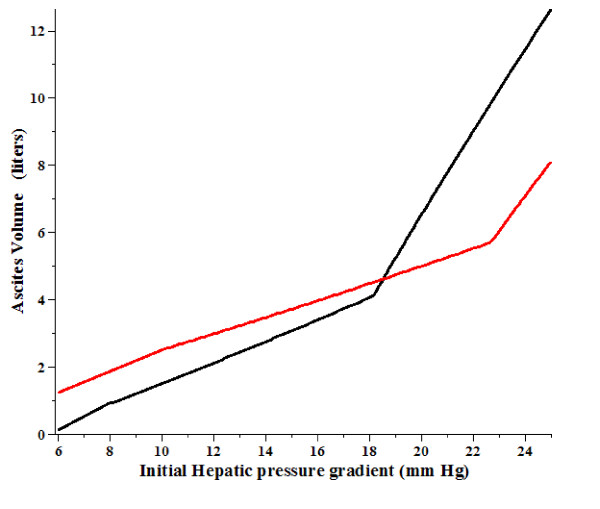
**Change in the ascites volume from the untreated case (black line) produced by a decrease in P_HVPG_by 20% and an increase in P_RA_from 5 to 7 mm Hg (red) as a function of the original P_HVPG_before treatment**.

As discussed in Section IIF, propranolol produces a decrease in the P_HVPG _similar to that of the diuretics, but it also produces an increase in P_Free _that results from an increase in P_RA_. Figure 4 shows the predicted change in ascites volume (red line) produced by a 20% decrease in P_HVPG _and an increase in P_RA _from 5 to 7 mm Hg, similar to what is seen for propranolol (Table 1). It can be seen that for initial P_HVPG _less than about 18 mm Hg, propranolol would be predicted to produce an increase in ascites volume, despite the decrease in P_HVPG_.

**Figure 5:**
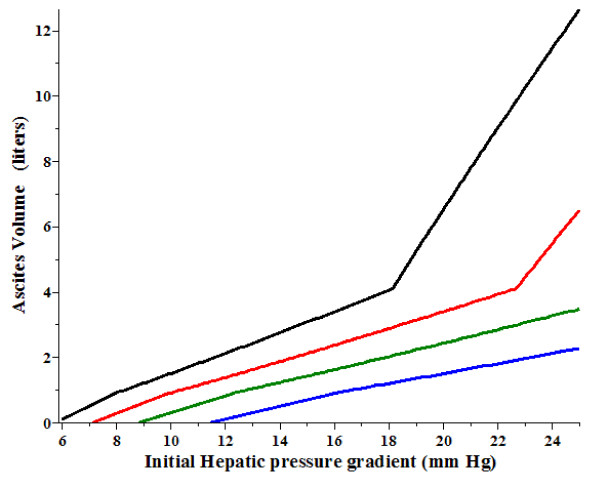
**Change in the ascites volume from the untreated case (black line) produced by reduction of either P_HVPG_or, equivalently, liver blood flow by 20% (red), 35% (green) or 50% (blue) as a function of the original P_HVPG_before treatment**.

A standard treatment for cirrhotic ascites is surgically produced portal systemic shunting [114]. Assuming that this procedure reduces portal flow with no change in liver resistance or central venous pressure, then the percent reduction in P_HVPG _is equal to the percent reduction in liver blood flow. Figure 5 shows the reduction in ascites volume produced by a 20%, 35% and 50% reduction in P_HVPG _or, equivalently, liver flow as a function of the initial P_HVPG_. It can be seen that in order to reduce ascites by shunting, large reductions (about 50%) in flow are required. This is consistent with the results of Rogriguez-Laiz et. al. [115] that showed that transjugular intrahepatic portasystemic shunts (TIPS) reduced liver blood flow by about 50%.

An important prediction of the model is that central venous pressure (P_RA_) plays a major role in ascites volume, independent of the P_HVPG_. For example, in a patient with an initial P_HVPG _of 16 mm Hg, a diuretic induced reduction in P_RA _of just 3 mm Hg would reduce ascites volume from 3.5 liters to a clinically undetectable 0.8 liters, with no change in P_HVPG_. Thus, the model is consistent with the clinical observation that that there may be only a loose correlation between the severity of ascites and P_HVPG_. It should also be pointed out that there need be no relationship between the initial P_HVPG _of subjects and their responsiveness to diuretics. For example a patient with an initial P_HVPG _of 16 mm Hg who cannot increase renal sodium output with diuretics (i.e., cannot reduce P_RA _or P_HVPG _in response to diuretics) will maintain an ascitic volume of about 3.5 liters. In contrast, a subject with an initial P_HVPG _of 20 mmHg who is responsive to diuretics, such that the P_RA _falls by 3 mmHg and the gradient by 4 mmHg, will have a reduction in ascitic volume from 6.5 liters to only 1 liter.

**Figure 6:**
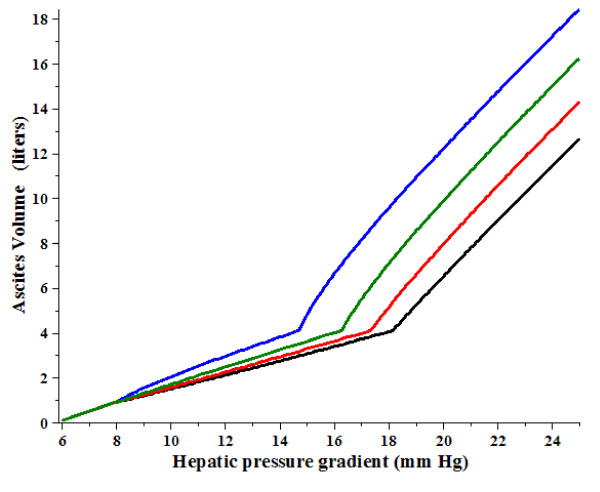
**Ascites volume as a function of P_HVPG_for a plasma colloid osmotic pressure of 25 mm Hg (black line), 20 mm Hg (red), 15 mm Hg (green), and 10 mm Hg (blue)**.

As discussed in Section IIF, it is expected that changes in plasma albumin should have a complicated effect on the severity of ascites. Figure 6 plots the predicted ascites volume as a function of P_HVPG _for plasma colloid osmotic pressure of 25 mm Hg (black line), 20 mm Hg (red), 15 mm Hg (green) and 10 mm Hg (blue). As expected (Section IIF), the increase in ascites volume produced by decreasing albumin is greatest at higher portal pressures and becomes negligible at P_HVPG _less than about 14 mm Hg. Even at P_HVPG _above14 mm Hg, the effect of decreasing oncotic pressure is relatively minor until this pressure reaches a value of 10 mm Hg (equivalent to a serum albumin of about 1.5 gm%). Thus, it is not surprising that many investigators have concluded that serum albumin infusion is not efficacious for the reduction of ascites. It also is apparent that no ascites would be expected with very low serum albumin concentrations, providing the P_HVPG _is normal (< 6 mm Hg). This prediction is borne out by the absence of ascites in subjects with congenital hypoalbumenemia [116,117].

**Figure 7:**
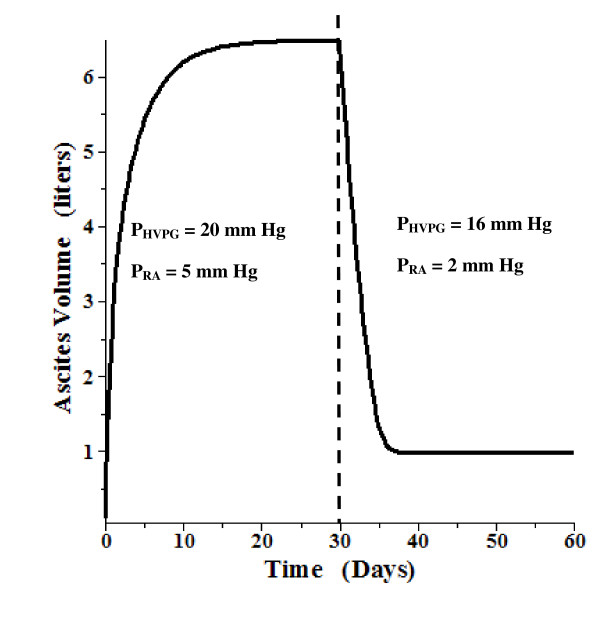
**Time dependent change in ascites volume**. At time 0, the P_HVPG _is raised to 20 mm Hg and P_RA _is raised to 5 mm Hg. After a steady state is reached at 30 days, P_HVPG _is reduced by 20% to 16 mm Hg and P_RA _is lowered to 2 mm Hg.

**Figure 8:**
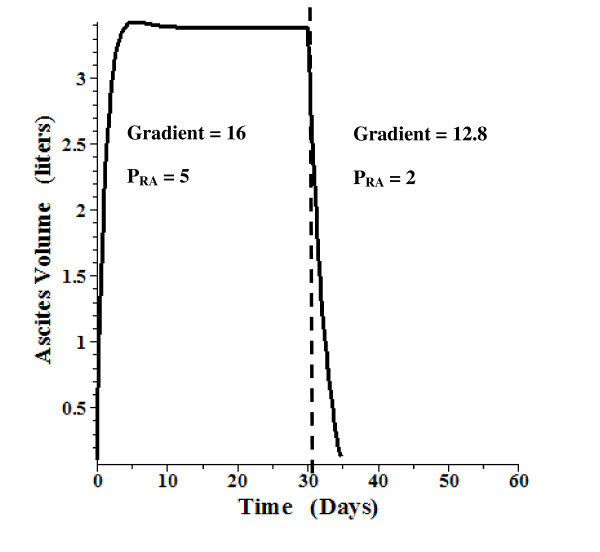
**Time dependent change in ascites volume**. At time 0, the P_HVPG _is raised to 16 mm Hg and P_RA _is raised to 5 mm Hg. After a steady state is reached at 30 days, P_HVPG _is reduced by 20% to 12.8 mm Hg and P_RA _is lowered to 2 mm Hg.

**Figure 9:**
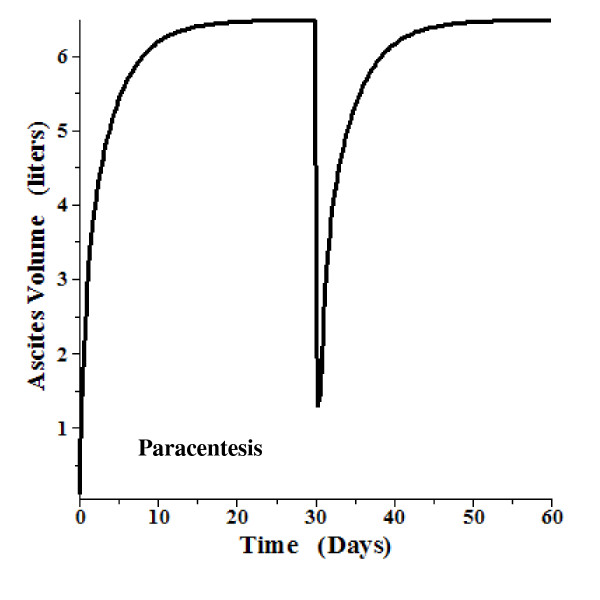
**Time dependent change in ascites volume**. At time 0, the P_HVPG _is raised to 20 mm Hg and P_RA _is raised to 5 mm Hg. After a steady state is reached at 30 days, 5.3 liters of ascites fluid is removed over a period of 12 hours and, over the next 10 days, the ascites reforms.

A time dependent model using the above relations and parameters was also developed. This is a detailed, general model which also includes the "intestinal" lymph flow and tissue compliance. The total "intestinal" hydraulic conductance (L_T_, eq. (18)) was divided equally between the intestinal capillaries and the intestinal mesothelium. (The following results do not depend significantly on this assumption). The solution requires the solution of 4 simultaneous differential equations. The details are described in the Additional file 1 Section II. Figure 7 shows the time dependent ascites volume change when, at time = 0, the gradient (P_HVPG_) is suddenly raised to 20 mm Hg and the right atrial pressure (P_RA_) is increased to 5 mm Hg. After 30 days, when the system has reached a steady state with a volume of 6.5 liters, the gradient is suddenly reduced by 20% (to 16 mm Hg) and P_RA _is reduced to 2 mm Hg. Over a period of about 5 days, the ascitic volume is reduced to about 1 liter. Figure 8 shows a similar plot, with the gradient raised to 16 mm Hg. A 20% reduction in gradient (and P_RA _reduced from 5 to 2) at 30 days leads to complete reabsorption of the ascites over a period of about 5 days. Figure 9 shows a plot of the volume change that might be expected following paracentesis. At t = 0 the gradient was raised to 20 mm Hg (P_RA _= 5) and the system reached a steady state at 30 days. Then, over a 12 hour period, 5.3 liters of ascitic fluid was removed following which the volume returned back to its initial volume over a period of about 5 days. The time dependence of these volume changes is similar to what is observed clinically [95].

We are unaware of any other attempts to quantitatively model ascites. The present model, which seemingly takes into account all factors involved in ascites accumulation, provides predictions that are in good accordance with the major clinical observations in ascites. However, it should be emphasized that the model employs assumptions that have only indirect experimental support. Two major assumptions are the quantitative linear pressure dependence of both the fluid leak from the liver (eq. (19)) and the peritoneal lymph flow (eq. (20)). Although the leak should be a function of (P_L _- P_A_), there is no evidence for the simple linear relation that is assumed. We are not aware of any measurements in humans that relate to the pressure dependence of the drainage of the peritoneal space. However, Zink and Greenway [94] showed in cats that the peritoneal fluid and protein absorption had an approximately linear dependence on pressure. Another important assumption in eq. (20) is that the lymph flow is sensitive to the central venous pressure (P_RA_). Although there are a number of observations that suggest that lymph flow is sensitive to thoracic duct pressure [28,118-121] and systemic venous pressure [122,123], these results are indirect and not conclusive.

### Summary

As discussed in the Introduction, in recent years ascites research largely has focused on the systemic changes observed in cirrhosis with particular emphasis on the "hyperdynamic circulation" syndrome. Although there is an implicit assumption that these changes cause ascites, there has been little discussion of the physiological link between these systemic alterations and ascites formation.

We theorize that ascites formation is dependent on just three factors: 1) the rate of protein leak from the liver, which is a function of the difference between the liver tissue pressure and the peritoneal pressure (P_L _- P_A_); 2) the colloid osmotic fluid movement between the intestinal tissue and the peritoneal space; and 3) the rate of lymph drainage of the peritoneal space, which is a function of the peritoneal pressure (P_A_) and the central venous pressure (P_RA_). This theory is not entirely novel in that other investigators [36,37,94,111] have recognized the role of these factors in ascites. Of particular importance is the work of Henriksen and colleagues that provides measurements of these factors in humans [33,47,92,93,111].

In the standard "Forward theory" of ascites formation, there is a cycle of pathological events, ending with sodium and water retention and plasma volume expansion which then produce the ascites [6]. The mechanism involved in this last step is either not discussed or assumed to be an obvious result of the volume expansion. In terms of the model we have presented, this volume expansion must act through an increase in central venous pressure (P_RA_) and/or an increase in liver tissue pressure (P_L_) produced by an increase in liver blood flow (as a result of increasing cardiac output).

The novel aspect of the present report is the development of a quantitative model of ascites accumulation based solely on physiologically relevant estimations of (P_L _- P_A_) and lymphatic drainage of the peritoneal cavity. The predictions of this model provide insight into a number of poorly understood clinical observations of the behavior of ascites in cirrhotic patients. For example, the model provides a quantitative explanation for the beneficial action of diuretics on ascites formation and the lack of efficacy of beta blockers despite their ability to reduce P_HVPG_. The model rather accurately predicts the known magnitude and time course of the action of diuretics on ascites. Lastly, the model provides a physiological explanation for the use of measurements of the SAAG to diagnose the existence of portal hypertension.

## Notation

Π_P_, Π_A_, Π_L_, Π_I_, colloid osmotic pressure in plasma, peritoneal fluid, and interstitial fluid of liver and intestine.; P_C_, P_A_, P_S_, P_L_, P_I_, P_PV_, P_HV_, P_RA_, P_Wedge_, P_Free_, P_HVPG_, hydrostatic pressure in intestinal capillary, peritoneal fluid, liver sinusoid, liver tissue, intestinal tissue, portal vein, hepatic vein, right atrium, the hepatic wedge and free pressures, and the hepatic vein pressure gradient (= wedge - free).; J_C_, J_I_, J_L_, J_lymph_, fluid flow rate across intestinal capillaries, intestinal mesothelium, liver capsule and peritoneal lymph flow.; L_C_, L_I_, L_T_, hydraulic conductivity across intestinal capillary, intestinal mesothelium and total.; F_L_, R_L_, liver blood flow and resistance.; L_y_, peritoneal lymph flow conductance.; P_min_, zero lymph flow pressure.; V_min_, peritoneal fluid volume when P_A _= P_min_; P_Break_, pressure at which liver capsule and/or lymphatics rupture.; m, protein concentration in fluid leaking from liver as fraction of plasma protein.

## Competing interests

The authors declare that they have no competing interests.

## Authors' contributions

DGL and MDL contributed equally to the manuscript. Both authors read and approved the final manuscript.

## Pre-publication history

The pre-publication history for this paper can be accessed here:

http://www.biomedcentral.com/1471-230X/12/26/prepub

## Supplementary Material

Additional file 1 **Experimental support for model and details of time dependent model solution**.Experimental support for model and derivation of time dependent model solution. Click here for file
