# Supplementary material for: Quantitative modeling of the physiology of ascites in portal hypertension
Source: BMC Gastroenterol. 2012 Mar 27;12:26. doi: 10.1186/1471-230X-12-26 (PMC3361476; doi:10.1186/1471-230X-12-26)
Supplement: Additional file 1 — Experimental support for model and details of time dependent model solution.Experimental support for model and derivation of time dependent model solution. [file 1471-230X-12-26-S1.DOC]

**Additional file 1. Experimental support for model and details of time dependent model solution.**

**I. Experimental support for the model.** (Note: all equation numbers in this section refer to equations in the main paper)
 **A. Intestinal capillary fluid balance.** It is assumed in the model that, as the portal vein and, therefore, the intestinal capillary pressure is raised, there is a corresponding washout of intestinal tissue protein which resets the Starling fluid balance across the capillary. Experimental quantitation of the terms in the Starling relation eq. 1 is difficult, requiring either direct micropuncture or indirect measurements of the pressure in the intestinal tissue and in the 5 micron diameter capillaries. In addition, this equation represents a steady state average balance of forces in a heterogeneous tissue, with different capillaries having different weighting. Finally, the capillary flows and pressures are very sensitive to local tissue conditions and the act of isolating and manipulating the tissue disturbs this steady state. Nevertheless, the few available measurements qualitatively support the model assumptions. Johnson and Richardson [1] measured the lymph flow, capillary pressure (isogravimetric technique), tissue pressure (micropuncture), tissue protein and colloid osmotic pressure (from lymph protein) as a function of venous pressure in the dog small intestine. As the venous pressure was raised from 0 to 15 mm Hg, the capillary pressure rose from 10 to 18 mm Hg, the lymph flow increased by a factor of 5, and the tissue colloid osmotic pressure decreased from 15 to 7 mm Hg as a result of tissue expansion and lymph flow washout. Raising the venous pressure from 0 to 25 mm Hg, decreased the tissue protein concentration from 5.1 to 1.0 g/100 gm. The tissue pressure remained 0 at all the venous pressures and the plasma colloid osmotic pressure was about 21 mm Hg. Using these experimental measurements in eq. 2 yields a net driving force of from 3 to 5 mm Hg over the entire range of venous pressures. This value is greater than is predicted in the above model for a net driving force ≈ 0 (eq. 3) and Johnson and Richardson [1] discuss various experimental errors that could explain this. In humans, the normal plasma oncotic pressure (Π_P_) is about 25 mm Hg and the normal intestinal lymph/plasma protein ratio is surprisingly high, about 0.7 [2, 3], corresponding to a Π_I_ =0.7*25 = 17.5 mm Hg. Assuming a value for the normal intra-abdominal pressure (P_A_) = 2 mm Hg which is assumed to be equal to P_I_ (eq. 6), eq. 3 predicts an average intestinal capillary pressure of 9.5 mm Hg. This is consistent with the above isogravimetric measurements of Johnson and Richardson [1] at low portal vein pressures. Using micropuncture, Davis and Gore [4] directly measured capillary pressure in the smooth muscle and villi of the rat intestine as a function of venous pressure. At venous pressures of 10, 20 and 30, the villus capillary pressures were 17, 25 and 33 mm Hg and the muscle capillary pressures were 19, 26 and 33 mm Hg. These results indicate that at the high portal vein pressures that are present in cirrhosis, the intestinal capillary pressure is only about 3 mm Hg greater than the venous pressure.

**B. Intestinal mesothelial fluid transport.** One of the more controversial assumptions of the model is that the intestinal mesothelium has protein permeability properties similar to the capillary and Starling’s relation (eq. 5) is valid for this membrane. There is large volume of literature in the peritoneal dialysis field describing and modeling the permeability properties of the “peritoneal” membrane. This literature suggests that the rate limiting membrane is the capillary, and that the mesothelium is highly permeable and leaky to proteins [5, 6]. Support for this assumption came from in vitro measurements performed on sheets of mesentery consisting of two mesothelial layers. Early measurements indicated that the mesentery was very leaky and had permeability properties that could not be distinguished from simple free aqueous diffusion [7, 8]. However, more recent measurement that have taken care to preserve tissue integrity have found that the mesothelial layers present a significant diffusive barrier and have permeability properties similar to the capillary endothelium [9-11]. There is direct evidence of a mesothelial barrier in the frog where electrical measurements indicate that the K^+^ resistance of the mesotheium is about 12 times greater than that of the capillary [12]. Additional support for a mesothelium barrier comes from recent measurements that show that aquaporin-1 knockout mice have a peritoneal water permeability about half that of normal mice [13] and that aquaporin is localized in mesothelial cells and its expression is upregulated by osmotic agents such as glucose or mannitol [14]. Finally, in our opinion, the strongest evidence for the presence of a significant mesothelial barrier is the implications of its absence. In its absence, the entire extravascular space of the intestine would simply be an extension of the peritoneal space. This is not consistent with the measurements discussed above which show a rapid washout of interstitial protein and an increase in intestinal lymph flow following a sudden increase in capillary pressure, or the increase in bowel wall thickness and edema that is characteristic of portal hypertension [15-17]. In the absence of a mesothelial barrier, increased portal vein pressure should result in fluid exudates from the intestinal surface, which are not observed [18].

**C. Liver sinusoidal fluid transport.** It is assumed in the model that the normal sinusoids do not restrict protein so that the tissue (and liver lymph) protein should equal the plasma value. This is supported by measurements that show that in normal dogs the experimental lymph/plasma is about 0.9 falling slightly (0.83) with large increases in sinusoidal pressure [2, 3] and, in cats, the normal value is about 0.8, which rises to 1.0 when hepatic pressure is raised to 10 mm Hg [19]. The protein size selectivity in the cat corresponds to a sinusoidal pore radius of about 18-25 nm at normal pressures, and greater than 100 nm at elevated pressures [19], much larger than the albumin gyration diameter of about 5.6 nm [20]. Direct EM measurements indicate a pore radius of 75- 87 nm [21]. The finding that the plasma/hepatic lymph ratio is slightly less than 1.0 could be partially explained by the contribution of lymph from the peribiliary capillaries (fig. 1). Since these capillaries restrict protein permeation, admixture of paracapillary lymph with sinusoidal lymph would lower the plama/lymph protein ratio of hepatic lymph. This effect is likely to be small since peribiliary blood flow represents only about 1/3 of the hepatic artery flow [22] and in a detailed review of the liver lymphatics, Trutmann [23] estimates that the peribiliary lymph contributes less than 10% of total hepatic lymph flow
 These results in normal animals generally support the model’s assumption of highly leaky sinusoids, with tissue protein nearly equal to plasma (lymph/protein of 0.8 to 1). In cirrhotic animals, there is a consistent observation of a decrease in the liver lymph/plasma ratio to a value of about 0.7 in both rats [24] and humans [3]. This is usually interpreted as a result of the “capillarization” of the sinuosids with with loss of the normal fenestra that occurs in cirrhosis [21, 25]. Although this 0.7 ratio is significantly less than the assumed model lymph/plasma ratio of 1.0, this discrepancy requires only minor modifications and does not significantly change the model’s implications. In particular, it does not alter the assumption that liver tissue and lymph fluid have high protein concentrations relative to ascitic and intestinal tissue, and the leak of this protein into the peritoneal space is the primary event in ascites formation.

**D. Source of ascitic protein – “weeping” from liver surface.** The main assumption of the ascites model is that the liver capsule and/or lymphatics rupture at a critical pressure, spilling the high protein liver tissue fluid into the peritoneal space. It is a classic observation that constriction of the inferior vena cava leads to obvious “weeping” of fluid droplets from the liver surface [18, 26, 27] while the other visceral surfaces appear dry [18]. Hyatt et. al. [18] collected 5 to 10 ml of fluid exuding from the liver over a several minute period. This fluid had a protein concentration nearly identical to that of plasma. Greenway and Laut [27] also found that this fluid had a specific gravity similar to plasma. Brauer et. al. [28] collected liver transudate from the rat liver as the hepatic vein pressure was raised. The transudate started when the hepatic vein pressure was raised about 3 mm Hg above normal, and increased rapidly with increasing pressure. The protein concentration in the transudate was identical to that in plasma. Clinical evidence that the weeping liver is the source of ascites protein is provided by the observation of Dumont and Mulholland [29] that “Lymph leaking from clusters of bulging lymphatics on the liver capsule and at the porta hepatis often is encountered at laparotomy in patients with Laennec’s cirrhosis”. Kuntz and Kuntz [30] provide a dramatic image of “Numerous, partially ruptured lymphocysts … on the liver surface with extravasation of protein-rich lymph in alcoholic cirrhosis” (fig. 16.5, p. 298). Tameda et. al. [31] observed “small lymphatic vesicles” on the liver surface in 65 out 372 cirrhotic subjects during peritoneoscopy. During laparoscopy, Heit et. al. [32] reported that 4 of 10 cirrhotic livers had surface “…lymphatic blebs indicating dilated lymphatic channels … and all 4 of these cases were complicated by ascites. Blebs were not seen in the absences of ascites.” While it is clear that a high protein fluid may weep from the liver and this weepage is associated with ascites, this does not exclude other sources of ascitic fluid. Quantitative evidence of the hepatic origin versus intestinal origin of ascites is provided by the remarkable experiments of Zimmon et. al. [33] in cirrhotic patients. They simultaneously labeled circulating albumin with ^131^I and the newly synthesized liver albumin with ^14^C-carbonate and demonstrated that most, if not all, of the ascites albumin derived directly from the liver.

The details of the leakage of fluid through the capsule are poorly understood. Even the histology of the interstitial space (e.g. space of Disse) and the lymphatics is controversial [23]. There is no question that increased sinusoidal pressure results in dilated interstitial spaces and superficial lymphatics [18, 26, 31, 32], but it is not known how these spaces drain into the peritoneal space. It has been proposed that the leak may occur when the lymphatics exit from the liver in the porta hepatis and are first exposed to the large pressure difference between the liver interstitial fluid and the intra-abdominal space [29].

**E. Ascites: inferior vena cava versus portal vein occlusion.** An important experimental observation is that formation in portal hypertension occurs only when there is an elevation of liver sinusoidal pressure and is not associated with just an increase in portal vein pressure [34]. This observation provides support for the model prediction that, within limits, increased portal pressure will washout intestinal tissue protein, establishing a new fluid balance equilibrium without the formation of ascites. This balance will break down and ascites may form only when the portal vein pressure becomes greater than the plasma colloid osmotic pressure (about 25 mm Hg) (assuming a negligible ascitic fluid pressure), a pressure that is seldom achieved in portal hypertension. The most dramatic confirmation of this resistance of the intestine to ascites formation was provided by the attempts of Witte et. al. [2] to produce ascites in dogs by either chronic inferior vena cava (IVC) constriction or by a combination of aorto-portal vein shunt and portal vein constriction, which produces very high portal vein pressures and normal sinusoidal pressure. As predicted, increases in sinusoidal pressure secondary to IVC constriction produced large amounts of ascites (400 ml) with only small increases in portal vein pressure of 11 mm Hg. In contrast, in the absence of elevated sinusoidal pressure, portal pressures of 18 mm Hg produced detectable ascites in only 5 of the 15 dogs and the amount of the ascites was small (45 ml). Portal pressures had to be raised to very high values (26 mm Hg) before large amounts of ascites (250 ml) were detected.

**F. Equilibration of the colloid osmotic pressure between intestinal tissue and ascites fluid.** The basic idea of the model is that the high protein fluid leaking from the liver into the peritoneal space pulls water osmotically from the non-liver visceral (e.g. intestine) interstitial space, lowering the peritoneal protein concentration. Quantitatively, this means that the colloid osmotic pressure of the ascitic fluid should be similar to that in the intestinal interstitial fluid which is a function of intestinal capillary and portal vein pressure. The experiments of Witte et. al. [2] discussed above provide direct confirmation of this hypothesis. For the IVC constriction case, the portal vein pressure was relatively low (11 mm Hg) and there was negligible washdown of intestinal interstitial protein. As a result, intestinal lymph protein (assumed equal to intestinal interstitial) was high (lymph/plasma = 0.55) and nearly identical to the ascites protein (lymph/plasma = 0.61). For the aorto-portal vein shunt and portal vein constriction case with a portal vein pressure of 26 mm Hg, tissue protein was washed down to very low values with a intestinal lymph/protein of 0.13 which, again, was nearly identical to the ascitic protein (lymph/protein = 0.16). In both cases, liver lymph protein remained high (lymph/protein ≈ 0.86). Witte et. al. [3] also measured ascitic and intestinal lymph/plasma protein ratios in late stage cirrhotic humans and found very low and nearly identical values for the intestinal (= 0.08) and ascitic (= 0.13) lymph/plasma ratios. Direct evidence of this colloid osmotic induced movement of fluid across the intestinal mesothelium is provided by measurements of peritoneal volume change when blood or plasma is placed in the rat peritoneal space [35, 36]. As predicted, there is an initial movement of fluid into the peritoneal space, diluting the fluid until the colloid osmotic pressure equilibrates, followed by slow absorption due to the intra-abdominal lymphatics.

**II. Details of time dependent solution.**

The same notation, definitions and relations that were used in Section IV to describe the steady state liver leak (J_L_), the peritoneal lymph flow (J_Lymph_) and the peritoneal pressure/volume relation (V(t)) are used here:

$$J_{L} = L_{L} (P_{L} - P_{A} - P_{Break})$$

$$\begin{matrix} J_{Lymph} = L_{Y}( P_{A} - P_{RA}+ P_{\min}) P_{A} > P_{RA} - P_{\min} \\ = 0 P_{A} < P_{RA} - P_{\min} \end{matrix}$$

$$V(t) = V_{\min} + D*[P_{A} - P_{\min}]$$

The rate of protein leak from the liver is equal to mΠ_P_ J_L_. Separate equations are used to describe the intestinal capillaries and intestinal mesothelium fluid flux, with the total intestinal hydraulic resistance = L_T_ ==L_I_L_C_/(L_C_+L_I_):

$$J_{C } = L_{C}[(P_{C}-P_{I}) - (\prod_{P}-\prod_{I})]$$

$$J_{I } = L_{I}[(P_{I}-P_{A}) - (\prod_{I}-\prod_{A})]$$

It is again assumed that the liver pressure = P_L_ =(P_P_+P_HV_)/2, the intestinal capillary pressure P_C_ is 3 mm Hg greater than the portal vein pressure P_P_ and that the hepatic vein pressure P_HV_ is either a) 2 mm greater than the right atrial pressure (P_RA_) in the absence of gross ascites; or b) equal P_A_ if P_A_ is greater than (P_RA_ +2):

$$\begin{matrix} P_{C} = P_{RA} + P_{HPVG} + 5 P_{A} < P_{RA} +2 \\ = P_{A} + P_{HPVG} +3 P_{A}>P_{RA} + 2 \end{matrix}$$

It is necessary to add a detailed model of the intestinal tissue space (protein concentration = Π_I_, pressure = P_I_, volume = V_I_, all now functions of time t).

$$\begin{matrix} P_{I}(t) = P_{A} + (V_{\mathrm{I}2} - V_{I1}) /D_{I} V_{I}(t) <= V_{I2} \\ = P_{A} + (V_{I}(t) - V_{I1})/D_{I} V_{I}(t)>V_{I2} \end{matrix}$$

where it is assumed that D_I_ = 133 ml/mm Hg, V_I2_= 50, V_I1_ = 100 ml. This relation allows the tissue pressure to have a maximum negative pressure relative to the peritoneal pressure P_A_ of (P_I_(t) – P_A_) = (V_I2_-V_I1_)/D_I_ at V_I_ = V_I2_. The intestinal lymph flow is described by:

$$\begin{matrix} J_{Ilymph} = 0 P_{I} < P_{A} \\ = L_{YI} (P_{I} - P_{A}) P_{I} > P_{A} \end{matrix}$$

Finally, a small rate of protein leak across the capillary (J_CA_) is added:

$$J_{CA} = Perm (\Pi_{P} - \Pi_{I})$$

Four differential equations are required to describe the intestinal volume V_I_(t), intestinal protein amount Amt_I_(t), peritoneal volume V(t) and peritoneal protein amount Amt_A_(t):

$$\frac{dV}{dt} = J_{I} +J_{L} - J_{Lymph}$$

$$\frac{d Amt_{A}}{dt} = m \Pi_{P} J_{L} - \Pi_{A} J_{Lymph}$$

$$\frac{d V_{I}}{dt} = J_{C} - J_{Ilymph} - J_{I}$$

$$\frac{d Amt_{I}}{dt} = J_{CA} - \Pi_{I}J_{Ilymph}$$

These equations were solved using the Maple (Maplesoft) solver and the time dependent peritoneal volume (V(t)) is plotted in figs. 7 -9. For these plots, the parameters derived for the steady state case were used: L_Y_ = 7.86, L_L_= 10.3, and L_T_ =6.25 ml/hour/mm Hg; P_Break_ =8, P_min_ = 2 mm Hg, D = 0.8 liters/mm Hg; and m = 0.8. It is assumed that the total L_T_ was divided equally between L_C_ and L_I_  (L_I_ = L_C_ = 2L_T_). In addition, the following new intestinal parameters were assumed: L_YI_ = 18 ml/lhour/mm Hg, D_I_ = 133 ml/mm Hg, V_I2_= 50, V_I1_ = 100 ml and Perm = 2 ml/hour. The peritoneal volume solution has only a very weak dependence on these intestinal parameters and they do not significantly alter the results in figs. 7 - 9.

**References:**

1. Johnson PC, Richardson DR: **The influence of venous pressure on filtration forces in the intestine**. *Microvasc Res* 1974, **7**(3):296-306.

2. Witte CL, Myers JF, Witte MH, Katz MA: **Transcapillary water and protein flux in the canine intestine with acute and chronic extrahepatic portal hypertension**. *Circulation research* 1983, **53**(5):622-629.

3. Witte CL, Witte MH, Dumont AE, Frist J, Cole WR: **Lymph protein in hepatic cirrhosis and experimental hepatic and portal venous hypertension**. *Annals of surgery* 1968, **168**(4):567-577.

4. Davis MJ, Gore RW: **Capillary pressures in rat intestinal muscle and mucosal villi during venous pressure elevation**. *The American journal of physiology* 1985, **249**(1 Pt 2):H174-187.

5. Flessner MF: **Osmotic barrier of the parietal peritoneum**. *The American journal of physiology* 1994, **267**(5 Pt 2):F861-870.

6. Rippe B, Rosengren BI, Venturoli D: **The peritoneal microcirculation in peritoneal dialysis**. *Microcirculation* 2001, **8**(5):303-320.

7. Nagel W, Kuschinsky W: **Study of the permeability of the isolated dog mesentery**. *European journal of clinical investigation* 1970, **1**(3):149-154.

8. Rasio EA: **Metabolic control of permeability in isolated mesentery**. *The American journal of physiology* 1974, **226**(4):962-968.

9. Agostoni E, Bodega F, Zocchi L: **Equivalent radius of paracellular "pores" of the mesothelium**. *J Appl Physiol* 1999, **87**(2):538-544.

10. Breborowicz A, Rodela H, Knapowski J, Oreopoulos DG: **Permeability of different parts of the peritoneal mesothelium to solutes: an in vitro study**. *Perit Dial Int* 1989, **9**(2):135-141.

11. Simon M: **Peritoneal mesothelium in vitro: an electrophysiologic study**. *Perit Dial Int* 1996, **16**(4):393-397.

12. Frokjaer-Jensen J, Christensen O: **Potassium permeability of the mesothelium of the frog mesentery**. *Acta physiologica Scandinavica* 1979, **105**(2):228-238.

13. Yang B, Folkesson HG, Yang J, Matthay MA, Ma T, Verkman AS: **Reduced osmotic water permeability of the peritoneal barrier in aquaporin-1 knockout mice**. *The American journal of physiology* 1999, **276**(1 Pt 1):C76-81.

14. Lai KN, Li FK, Lan HY, Tang S, Tsang AW, Chan DT, Leung JC: **Expression of aquaporin-1 in human peritoneal mesothelial cells and its upregulation by glucose in vitro**. *J Am Soc Nephrol* 2001, **12**(5):1036-1045.

15. Guingrich JA, Kuhlman JE: **Colonic wall thickening in patients with cirrhosis: CT findings and clinical implications**. *Ajr* 1999, **172**(4):919-924.

16. Karahan OI, Dodd GD, 3rd, Chintapalli KN, Rhim H, Chopra S: **Gastrointestinal wall thickening in patients with cirrhosis: frequency and patterns at contrast-enhanced CT**. *Radiology* 2000, **215**(1):103-107.

17. Marshak RH, Khilnani M, Eliasoph J, Wolf BS: **Intestinal edema**. *The American journal of roentgenology, radium therapy, and nuclear medicine* 1967, **101**(2):379-387.

18. Hyatt RE, Lawrence GH, Smith JR: **Observations on the origin of ascites from experimental hepatic congestion**. *The Journal of laboratory and clinical medicine* 1955, **45**(2):274-280.

19. Granger DN, Miller T, Allen R, Parker RE, Parker JC, Taylor AE: **Permselectivity of cat blood-lymph barrier to endogenous macromolecules**. *Gastroenterology* 1979, **77**(1):103-109.

20. Kiselev MA, Gryzunov Iu A, Dobretsov GE, Komarova MN: **[Size of a human serum albumin molecule in solution]**. *Biofizika* 2001, **46**(3):423-427.

21. Braet F, Wisse E: **Structural and functional aspects of liver sinusoidal endothelial cell fenestrae: a review**. *Comparative hepatology* 2002, **1**(1):1.

22. Takasaki S, Hano H: **Three-dimensional observations of the human hepatic artery (Arterial system in the liver)**. *Journal of hepatology* 2001, **34**(3):455-466.

23. Trutmann M, Sasse D: **The lymphatics of the liver**. *Anatomy and embryology* 1994, **190**(3):201-209.

24. Barrowman JA, Granger DN: **Effects of experimental cirrhosis on splanchnic microvascular fluid and solute exchange in the rat**. *Gastroenterology* 1984, **87**(1):165-172.

25. Henriksen JH, Horn T, Christoffersen P: **The blood-lymph barrier in the liver. A review based on morphological and functional concepts of normal and cirrhotic liver**. *Liver* 1984, **4**(4):221-232.

26. Bolton C, Barnard WG: **The pathological occurences in the liver in experimental venous stagnation**. *The Journal of Pathology and Bacteriology* 1931, **34**(6):701-709.

27. Greenway CV, Lautt WW: **Effects of hepatic venous pressure on transsinusoidal fluid transfer in the liver of the anesthetized cat**. *Circulation research* 1970, **26**(6):697-703.

28. Brauer RW, Holloway RJ, Leong GF: **Changes in liver function and structure due to experimental passive congestion under controlled hepatic vein pressures**. *The American journal of physiology* 1959, **197**:681-692.

29. Dumont AE, Mulholland JH: **Alterations in Thoracic Duct Lymph Flow in Hepatic Cirrhosis: Significance in Portal Hypertension**. *Annals of surgery* 1962, **156**(4):668-675.

30. Kuntz E, Kuntz H: *Hepatology textbook and atlas: history, morphology, biochemisty, diagnostics, clinic, therapy*. 3 edition. Heidelberg: Springer; 2008.

31. Tameda Y, Yoshizawa N, Takase K, Nakano T, Kosaka Y: **Prognostic value of peritoneoscopic findings in cirrhosis of the liver**. *Gastrointestinal endoscopy* 1990, **36**(1):34-38.

32. Heit HA, Johnson LF, Rabin L: **Liver surface characteristics as observed during laparoscopy correlated with biopsy findings**. *Gastrointestinal endoscopy* 1978, **24**(6):288-290.

33. Zimmon DS, Oratz M, Kessler R, Schreiber SS, Rothschild MA: **Albumin to ascites: demonstration of a direct pathway bypassing the systemic circulation**. *The Journal of clinical investigation* 1969, **48**(11):2074-2078.

34. Schilling JA, Mc CA, Clausen SW, Troup SB, Mc KF: **Experimental ascites; studies of electrolyte balance in dogs with partial and complete occlusion of the portal vein and of the vena cava above and below the liver**. *The Journal of clinical investigation* 1952, **31**(7):702-710.

35. Chen TW, Khanna R, Moore H, Twardowski ZJ, Nolph KD: **Sieving and reflection coefficients for sodium salts and glucose during peritoneal dialysis in rats**. *J Am Soc Nephrol* 1991, **2**(6):1092-1100.

36. Courtice FC, Harding J, Steinbeck AW: **The removal of free red blood cells from the peritoneal cavity of animals**. *The Australian journal of experimental biology and medical science* 1953, **31**(3):215-225.
